# Genetic mutation of *Cep76* results in male infertility due to abnormal sperm tail composition

**DOI:** 10.26508/lsa.202302452

**Published:** 2024-04-03

**Authors:** Brendan J Houston, D Jo Merriner, G Gemma Stathatos, Joseph H Nguyen, Anne E O’Connor, Alexandra M Lopes, Donald F Conrad, Mark Baker, Jessica EM Dunleavy, Moira K O’Bryan

**Affiliations:** 1 School of BioSciences and Bio21 Molecular Sciences and Biotechnology Institute, The University of Melbourne , Parkville, Australia; 2 Instituto de Investigação e Inovação em Saúde, Universidade do Porto, Porto, Portugal; 3 Institute of Molecular Pathology & Immunology, University of Porto, Porto, Portugal; 4 Oregon National Primate Research Center, Oregon Health and Science University, Beaverton, OR, USA; 5 School of Environmental and Life Sciences, The University of Newcastle, Callaghan, Australia; 6 School of Biomedical Sciences and Pharmacy, College of Health, Medicine and Wellbeing, The University of Newcastle, Callaghan, Australia

## Abstract

The transition zone is a specialised gate at the base of cilia/flagella, which separates the ciliary compartment from the cytoplasm and strictly regulates protein entry. We identified a potential new regulator of the male germ cell transition zone, CEP76. We demonstrated that CEP76 was involved in the selective entry and incorporation of key proteins required for sperm function and fertility into the ciliary compartment and ultimately the sperm tail. In the mutant, sperm tails were shorter and immotile as a consequence of deficits in essential sperm motility proteins including DNAH2 and AKAP4, which accumulated at the sperm neck in the mutant. Severe annulus, fibrous sheath, and outer dense fibre abnormalities were also detected in sperm lacking CEP76. Finally, we identified that CEP76 dictates annulus positioning and structure. This study suggests CEP76 as a male germ cell transition zone protein and adds further evidence to the hypothesis that the spermatid transition zone and annulus are part of the same functional structure.

## Abstract (precis)

This study explores the role of CEP76 in male fertility via its predicted role in establishing a functional transition zone that mediates protein entry into the sperm tail.

## Graphical Abstract

**Figure:**
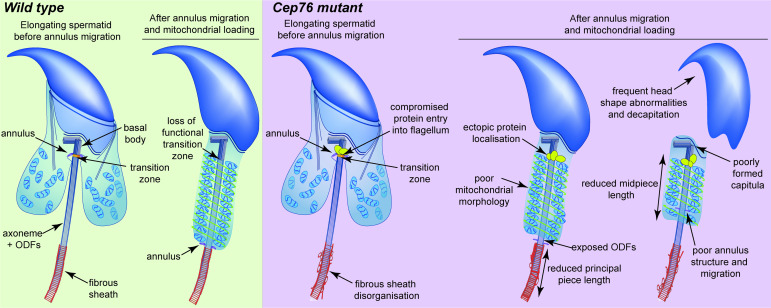

## Introduction

Cilia and their organelle cousins, flagella, play essential roles in many cell types. Notably, a single modified motile cilium (a flagellum) projects from male gametes and is essential for fertility in sexually reproducing animals (1). In eukaryotic cilia, the transition zone (TZ), also known as the ciliary gate, has emerged as an essential mediator of cilium development through its role in controlling protein entry into the ciliary compartment within which cilia/flagella develop (2, 3, 4). The TZ develops immediately distal to the mature centriole, from which the core of the cilia, the axoneme, develops. The TZ allows for the selective transport of proteins into the cilium/flagellum and thus the establishment and maintenance of a unique ciliary microenvironment (5, 6). In somatic cells, the TZ is largely composed of sheets of Y-shaped structures that attach at one side to the developing axoneme, at a single point, and at the other side dock to the plasma membrane, at two points (7, 8). Although TZ composition, including Y-shaped linkers, is poorly understood, several genes that when mutated result in ciliopathies have been identified as core members of the TZ (9, 10, 11, 12). To enact protein and vesicle transport across the TZ and along the developing axoneme, cilia/flagella use a bidirectional transport process of intraflagellar transport with the aid of the Bardet–Biedl syndrome complex (13, 14). The Bardet–Biedl syndrome is a protein complex of at least eight proteins that plays essential roles in facilitating cargo entry into, and exit from, the ciliary compartment. In addition, the distal appendages of the centriole (known as transition fibres) play an essential role in protein trafficking into the ciliary compartment by acting as docking sites for cargoes before their entry through the TZ (15).

The sperm tail is a modified motile cilium, and although it is assumed that the formation of the TZ and core of the axoneme will be similar to that which occurs in somatic cells, this is largely untested. Current models of the TZ are largely informed by data from primary cilia, or the flagella of lower order species such as *Chlamydomonas* (16). There are, however, several studies highlighting a functional TZ in *Drosophila* spermatids (17, 18) and emerging evidence to suggest differences in TZ structure, composition, and transport machinery exist to meet the demands of different cilium/flagellum subtypes (reviewed in reference 19). As mammalian sperm contain accessory structures not seen in somatic cells, for example, it is reasonable to predict that the male germ cell TZ is modified to selectively recruit fibrous sheath and outer dense fibre proteins into the ciliary lobe.

As with all motile cilia/flagella, sperm tail motility is dictated by the function of the axoneme—a 9 + 2 microtubule-based structure that runs the length of the tail (20, 21). The sperm tail is composed of two major sections: the midpiece, which houses the mitochondria; and the principal piece, wherein glycolytic enzymes are anchored to a structure called the fibrous sheath (22, 23, 24, 25). Both the midpiece and principal piece compartments also contain outer dense fibres—which are circumferential to the axoneme microtubules and act to protect the sperm tail from shearing forces (26, 27). In the principal piece, the longitudinal columns of the fibrous sheath replace the outer dense fibres 3 and 8 and are linked by circumferential ribs (28, 29). The outer dense fibres and fibrous sheath are formed within the ciliary compartment, and as such, their component proteins must transit through the TZ (26). Although this may suggest the requirement for additional factors to aid in the selective transport of sperm-specific proteins via the TZ into the ciliary compartment, no such proteins of this function have been identified.

In addition, towards the end of sperm tail development, the annulus, a septin-based ring that is thought to be attached to, or physically a part of the TZ, migrates distally along the sperm tail to define the junction of what will ultimately become the midpiece and principal pieces of the sperm tail (30, 31). The annulus is thought to be a diffusion barrier between the subcompartments of the tail, and its loss is often associated with a sharp bending at the midpiece–principal piece junction, poor sperm motility, and ultimately male infertility (30, 32). At a similar time to annulus migration, but by a seemingly independent process (30, 33, 34), the membrane attached to the basal body is pulled distally to the annulus, thus exposing a portion of the axoneme (surrounded by the outer dense fibres) to the cytoplasm. This process allows cytoplasmic mitochondria to be loaded onto the sperm tail to form the mitochondrial sheath of the midpiece (26, 35).

**Figure S1.**
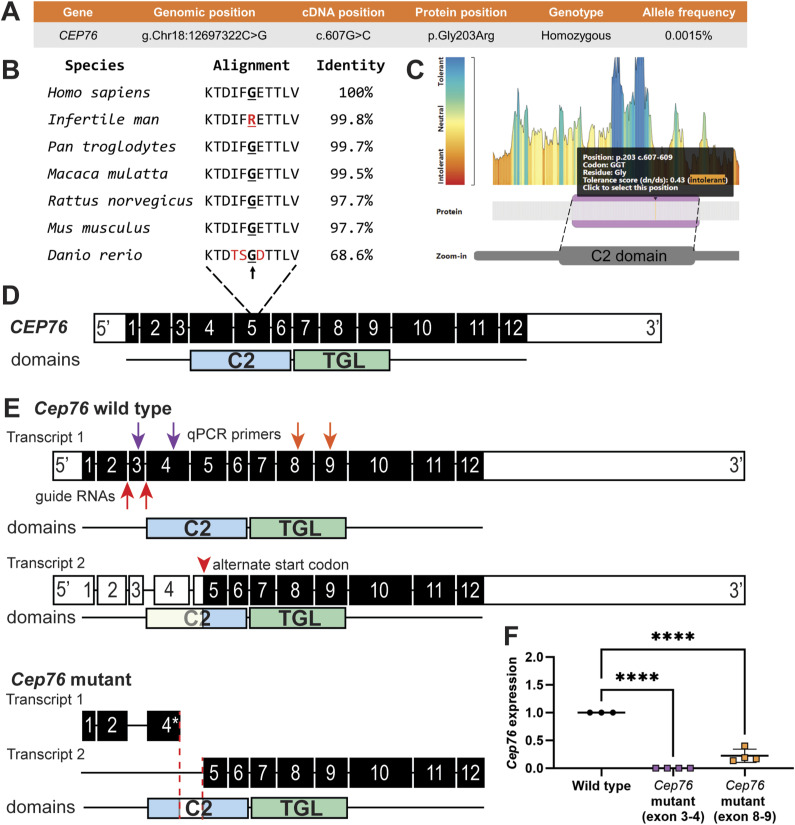
*CEP76* human genetic variant and *Cep76* mutant mouse model. **(A)** CEP76 mutation identified in an infertile man, including genetic coordinates and protein position. **(B)** CEP76 protein species alignment, including conserved amino acid (G, bolded + underlined), and total protein identity across species. Red letters denote amino acids not conserved in zebrafish (*Danio rerio*) and the affected amino acid in the infertile man. **(C)** MetaDome assessment of the affected amino acid and its tolerance to change, which was assessed as intolerant. **(D)** Genetic variant affected exon 5 of the human CEP76 protein, within the C2 (ciliary targeting, exons 4–6) domain. CEP76 in addition holds a TGL (transglutaminase, exons 7–9) domain. **(E)** Mouse full-length (and a second, shorter) *Cep76* transcript and *Cep76* mutant transcripts shown below (* denotes a premature stop codon in exon 4). Red arrows denote—where guide RNAs for exon 3 removal were targeted—intronic regions surrounding exon 3; purple and orange arrows denote approximate target sites of qRT–PCR primers. **(F)** *Cep76* expression as measured by qRT–PCR in wild-type and mutant testes, relative to housekeeper *Ppia* expression (n ≥ 3) and normalised to wild-type levels. ****P < 0.0001.

Despite our descriptive understanding of a number of these processes, the mechanisms of sperm tail development are still largely unknown. It is currently unclear how sperm-specific proteins, including components of the accessory structures, are selectively transported via the TZ into the ciliary compartment. In working to address this, we identified the previously unexplored centriole gene *CEP76* as being essential for male fertility in men as highlighted by a missense mutation in an infertile man with azoospermia (Fig S1A and D [36, 37]). CEP76 has been suggested to play a role in centriole duplication in somatic cell lines through interaction with the centriole protein CP110 (38, 39), but nothing was known about its role in male fertility. *CEP76* expression is testis-enriched (40), and CEP76 has been localised to centrioles in human spermatozoa and somatic cells (39, 41). CEP76 has two putative functional domains (Fig S1): a C2 domain that is predicted to function in ciliary membrane targeting, and a transglutaminase domain that is predicted to interact with tubulins in the axoneme or the TZ (42). It is hypothesised, based on a large TZ protein comparative study, that these domains cooperate to play a role in the Y-shaped linker function (42) and likely TZ function. Thus, we aimed to define the role CEP76 plays in male fertility.

Within this study, we identify CEP76 as an essential male fertility gene, with a role in TZ function and the selective entry of key motility proteins into the developing flagellum. *Cep76* mutant males were sterile and produced sperm with a variety of structural defects and the abnormal accumulation of key proteins at the sperm neck, consistent with an inability to pass through the TZ during the process of tail development. Consequently, the formation of the mitochondrial sheath was significantly impaired. Collectively, these data suggest CEP76 is the first known protein predicted to play a germ cell–specific role in regulating TZ function, and thus male fertility, in mammals.

## Results

### *Cep76* is a spermatid-enriched mRNA

**Figure S2.**
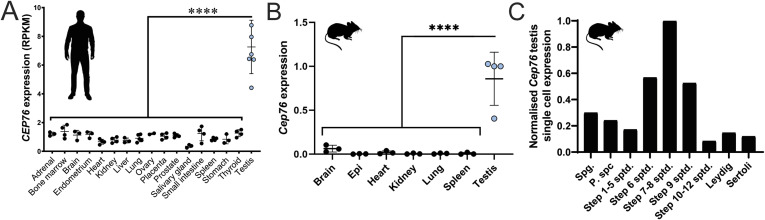
*Cep76* is a spermatid-enriched gene. **(A)** *CEP76* expression across human tissues as assessed by RNA-seq. **(B)** *Cep76* expression across major organs in mice as assessed by qRT–PCR relative to housekeeper *Ppia*. **(C)** *Cep76* expression in mouse testis cell types as defined by single-cell sequencing. Spg., spermatogonia; P spc., pachytene spermatocyte; sptd., spermatid.

Consistent with its putative role in male fertility, human *CEP76* and mouse *Cep76* are most prominently expressed in the testis (Fig S2A and B). To define which germ cell types *Cep76* is expressed in, we used single-cell RNA-sequencing data for mouse testes. This revealed that *Cep76* expression is considerably elevated in step 6–9 spermatids (Fig S2C), at the time when the core of the sperm tail is formed. We tested multiple antibodies to investigate CEP76 localisation in male germ cells but found all were non-specific (data not shown). All antibodies reacted with antigens in mutant tissue and at the incorrect size, suggesting they bound non-specifically.

### *Cep76* is required for male fertility in mice

To directly test the hypothesis that CEP76 is required for sperm tail development and thus male fertility, we generated a mutant mouse model using the CRISPR/Cas9 technology. We removed exon 3 of the mouse gene (Fig S1E), which resulted in a premature stop codon in exon 4 of *Cep76* of the full-length protein-coding transcript (ENSMUST00000097542.3). This transcript encodes a protein of identical length (659 AAs, predicted molecular mass of 74.3 kD) and 97.7% identity (Fig S1B) to the human CEP76 principal isoform. In addition, sequence comparison revealed that the amino acid affected in the infertile man is a highly conserved residue from men to zebrafish (Fig S1B and C). We note that *Cep76* is predicted to encode a second transcript that generates a smaller protein (54 kD) that only contains the complete TGL domain. This transcript is predicted to be unaffected by the genetic modification because of the presence of an alternative start codon in exon 5. The complete removal of exon 3 from all mRNA was confirmed via qRT–PCR (Fig S1F). Primers targeting mRNA downstream of the deletion revealed a highly significant reduction in mRNA in the mutant to 22% of wild-type levels. This residual mRNA may correspond to the expression of the second transcript. No functional protein was, however, predicted to be expressed.

**Figure 1.**
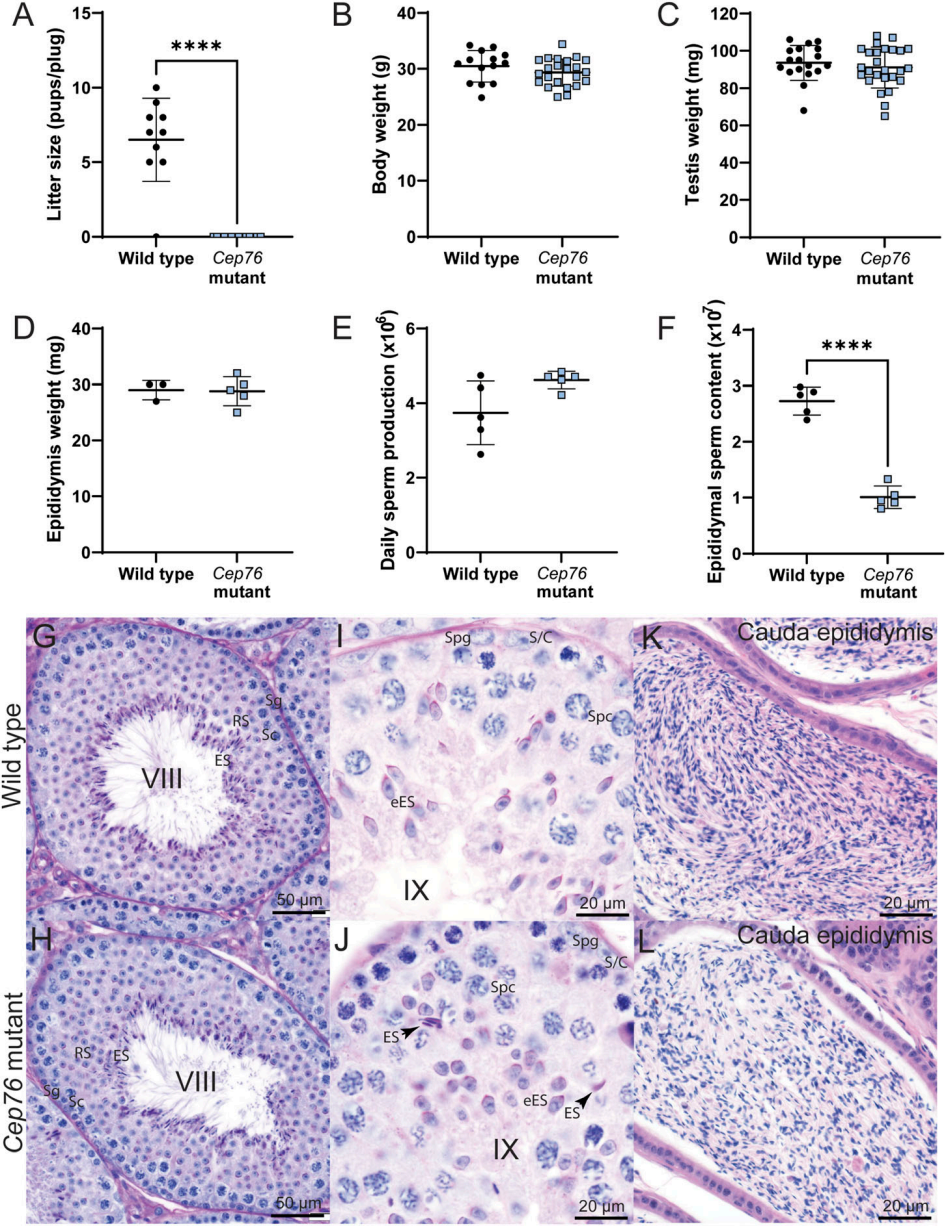
CEP76 is essential for male fertility. **(A)** Wild-type versus *Cep76* mutant data for (A) litter size (n = 10), **(B)** body weight (n ≥ 15), **(C)** testis weight (n ≥ 17), **(D)** epididymis weight (n ≥ 3), **(E)** daily sperm production per testis (n = 5), and **(F)** epididymal sperm content (n = 5). ****P < 0.0001. **(G, H, I, J)** Histology of wild-type and (H, J). *Cep76* mutant testis sections of stage VIII and stage IX tubules, respectively. Arrows in (J) point to retained spermatids as evidence of spermiation failure. Sg, spermatogonia; Sc, spermatocyte; RS, round spermatid; ES, elongated spermatid (eES, elongating spermatid); S/C, Sertoli cell. **(K, L)** Cauda epididymis sections are shown for wild type and *Cep76* mutant. Scale bars are noted to equal 50 or 20 μm.

Although mutant males were free of overt systemic disease and displayed normal mating behaviour, they were sterile (Fig 1A). Mutant females were fertile (not shown). Body, testis, and epididymis weights (Fig 1B–D) of *Cep76* mutant males were equivalent to wild-type males. Although the daily sperm production of *Cep76* mutant males was similar to wild-type counterparts (Fig 1E), the number of sperm within their epididymides was reduced to ∼35% of wild-type levels (Fig 1F; *P* < 0.0001). The reduced sperm number in the *Cep76* mutant epididymis, in the absence of a reduction in testis weight and daily sperm production, was due at least in part to spermiation failure, as evidenced histologically in stage IX tubules, wherein sperm were retained within the seminiferous epithelium (Fig 1J). Sperm were not observed in wild-type tubules at a comparable stage (Fig 1I). Other processes, including the selective removal of abnormal sperm from the epididymides of mutant males, may also have contributed to the reduced sperm output. With the exception of spermiation failure, spermatogenesis appeared normal at a light microscopic level in *Cep76* mutants (Fig 1H) and was comparable to wild type (Fig 1G). Similarly, epididymal histology was comparable between genotypes (Fig 1K and L).

### CEP76 is required for normal sperm morphology and motility

**Figure 2.**
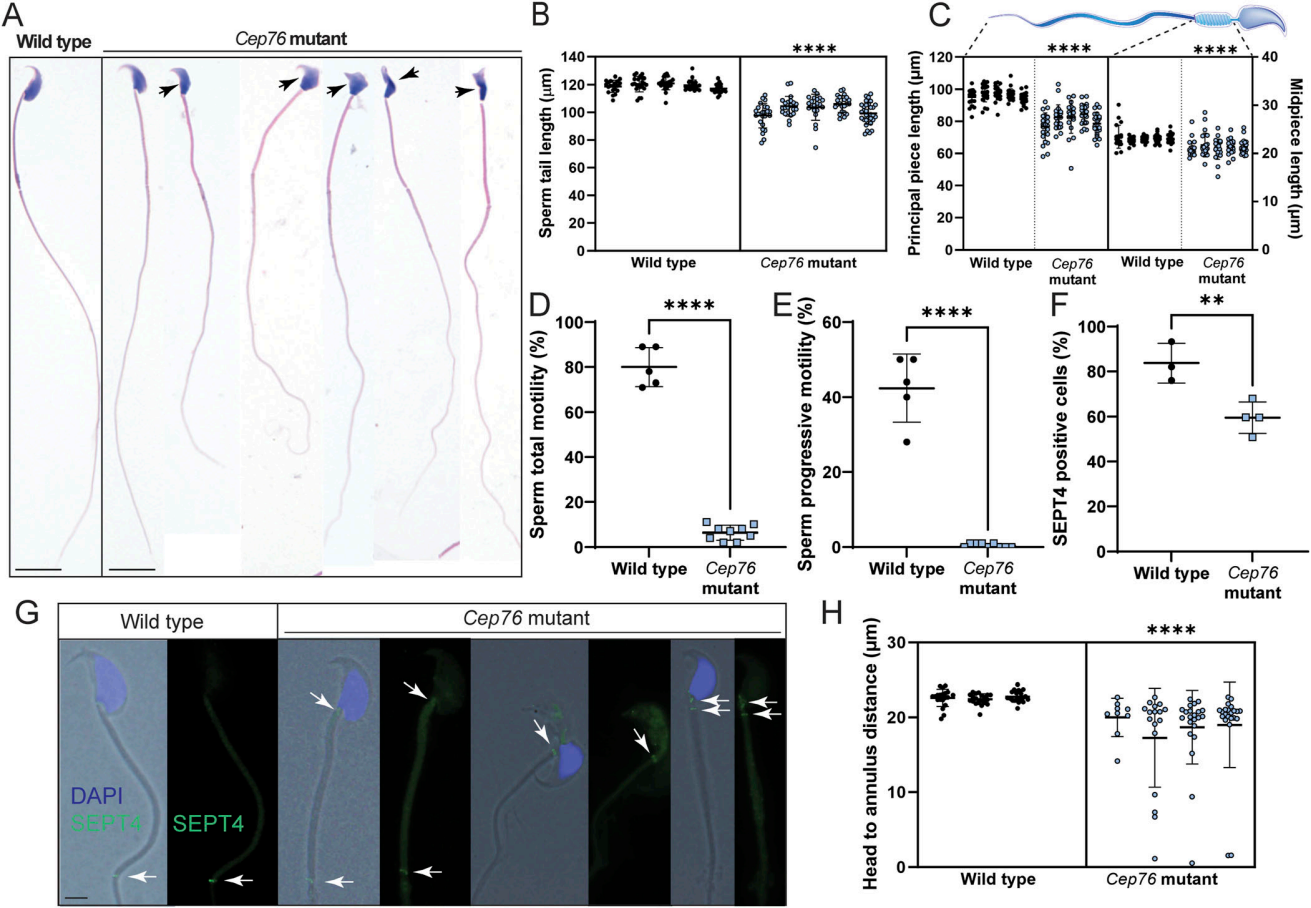
CEP76 is required for normal sperm morphology, motility, and tail length. **(A)** Wild-type versus *Cep76* mutant data for (A) sperm morphology, **(B)** total sperm tail length (n = 5), **(C)** sperm principal piece (left) and midpiece (right) length (n = 5), **(D, E)** total and (E) progressive sperm motility (both n ≥ 5), **(F)** incidence of SEPT4 staining in sperm, **(G)** sperm annulus staining (SEPT4; n ≥ 3) (scale bar = 2 μm), and **(H)** sperm annulus migration distance (n ≥ 3). **P < 0.01, ****P < 0.0001.

Analysis of sperm morphology via light microscopy revealed overt defects in sperm from mutant males (Fig 2A). Total sperm tail length, principal piece length, and midpiece length (measured by mitochondrial sheath staining) of sperm from *Cep76* mutant males were significantly shorter (by 15%, 16%, and 25%, respectively) than those from wild-type males (Fig 2B and C; all *P* < 0.0001), suggesting a fundamental role of CEP76 in tail assembly within the sperm ciliary compartment and mitochondrial loading. In agreement with the defects in sperm morphology, computer-assisted sperm analysis revealed a striking reduction in the percentage of sperm from mutants displaying any form of motility (Fig 2D; 6% in mutant versus 80% in wild type, *P* < 0.0001) and the virtual absence of forward, progressively motile sperm in comparison with wild-type males (Fig 2E; 0.4% in mutant versus 42.4% in wild type, *P* < 0.0001). The small population of sperm from *Cep76* mutant males that were classified as motile were seen to be simply twitching on the spot.

In addition, we measured the distance of annulus migration from the base of the sperm head, where SEPT4 staining was used to mark the annulus (Fig 2F–H). Measurements revealed a significantly shorter distance between the annulus and the nucleus in sperm from *Cep76* mutant males relative to wild type (18.6 μm in mutant versus 22.6 μm in wild type; *P* < 0.0001). In addition, the portion of sperm with SEPT4-positive staining (Fig 2F) was significantly reduced in the absence of CEP76 (60% in mutant versus 84% in wild type, *P* = 0.019). Of note in some sperm from *Cep76* mutants, we also identified ectopic SEPT4 staining at the sperm neck, suggestive of TZ localisation (Fig 2G). In the same sperm, SEPT4 was also still observed at the annulus. This was never seen in wild-type sperm and suggests CEP76 is required for both annulus formation and migration. It likely also suggests that the annulus is fragmented in sperm from *Cep76* mutants.

**Figure S3.**
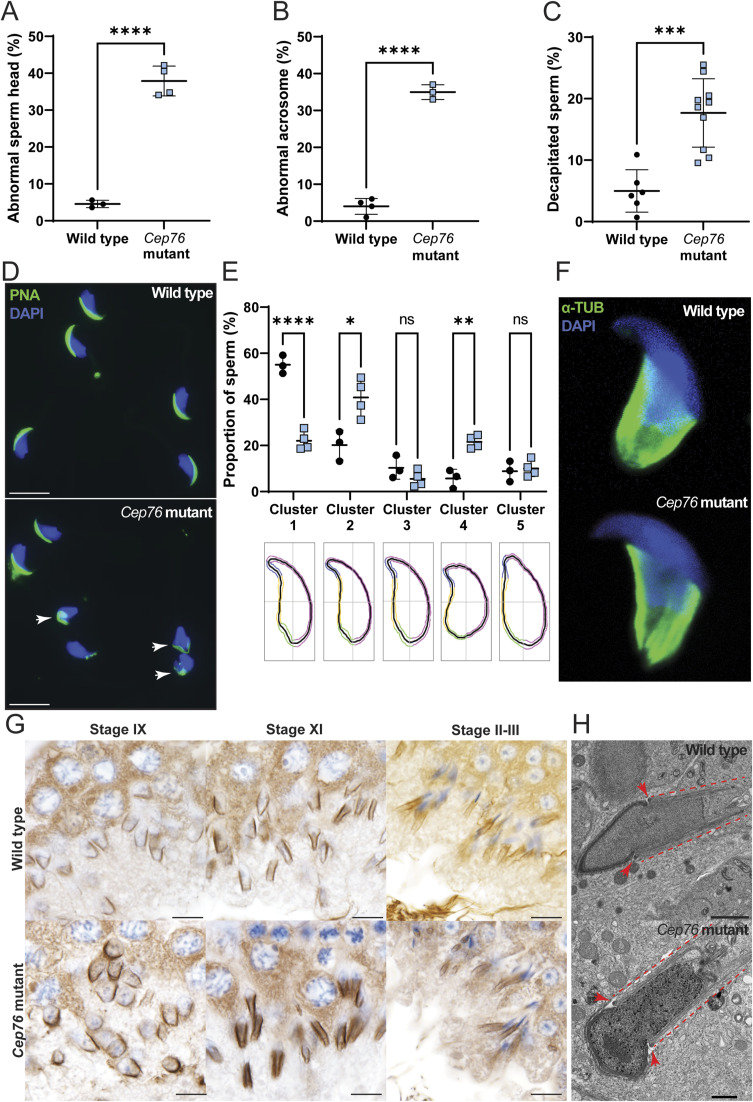
CEP76 is required for sperm head and acrosome shape but not normal manchette formation. **(A)** Abnormal sperm head shape assessment (n ≥ 3). **(B)** Abnormal sperm acrosome formation assessment (n ≥ 3). **(C)** Sperm decapitation assessment (n ≥ 5). **(D)** Sperm head and acrosome morphology assessment on cells stained with DAPI and PNA. **(E)** Objective sperm head shape analysis and the proportion of sperm falling into each cluster (n ≥ 3). Cluster 1 are most normal through to Cluster 5 as the most abnormal head shapes. Representative traces are shown for head shapes below each cluster. **(F)** Alpha-tubulin staining on isolated elongated spermatids to highlight the manchette (green) and sperm nucleus (blue). **(G)** Spermatids at stages IX, XI, and II-III stained with alpha-tubulin to mark the manchette are shown. **(H)** Transmission electron microscopy images of the manchette structure in elongating spermatids. Dashed lines indicate the manchette microtubules, and arrows denote the perinuclear ring. Scale bars are 10 μm in length, except for (H) where they are 1 μm in length. **P < 0.01, ****P < 0.0001.

Light microscopy also revealed defects in sperm head shape (Fig S3A) in at least 40% of sperm from knockouts compared with 5% in wild type (*P* < 0.0001). We also observed a significant increase in sperm with abnormal acrosomes from *Cep76* mutant males (35% in mutant versus 3% in wild type, Fig S3D; *P* = 0.0002). In addition, we analysed sperm head morphology using nuclear shape analysis software. This revealed a significantly lower proportion of the mutant population classified as normal (Cluster 1), although there were significant increases in sperm with slightly (Cluster 2) and largely (Cluster 4) abnormal head shapes (compared with wild type). This analysis revealed that ∼70% of sperm heads from *Cep76* mutant males had abnormal nuclear morphology (Fig S3E; *P* = 0.0002). In addition, there was a twofold increase in sperm decapitation in samples from mutant males, indicating that CEP76 plays a role in establishing patency of the head–tail coupling apparatus (HTCA) (Fig S3C; *P* = 0.0002).

Collectively, these results reveal CEP76 is required for the production and release of normal numbers of functional sperm. Shorter sperm tails and annulus defects suggest CEP76 plays a role in TZ function. Defects in nuclear morphology and a weakened HTCA are supportive of a role of CEP76 in manchette function and/or acrosome formation and in the fortification of the neck region (a derivative of the basal body) in late spermiogenesis. It is, however, currently unclear as to how CEP76 would achieve this role. At a functional level, sperm from *Cep76* mutant males are unable to reach the site of fertilisation because of highly impaired sperm motility.

### CEP76 is required for normal sperm flagella and HTCA ultrastructure

**Figure 3.**
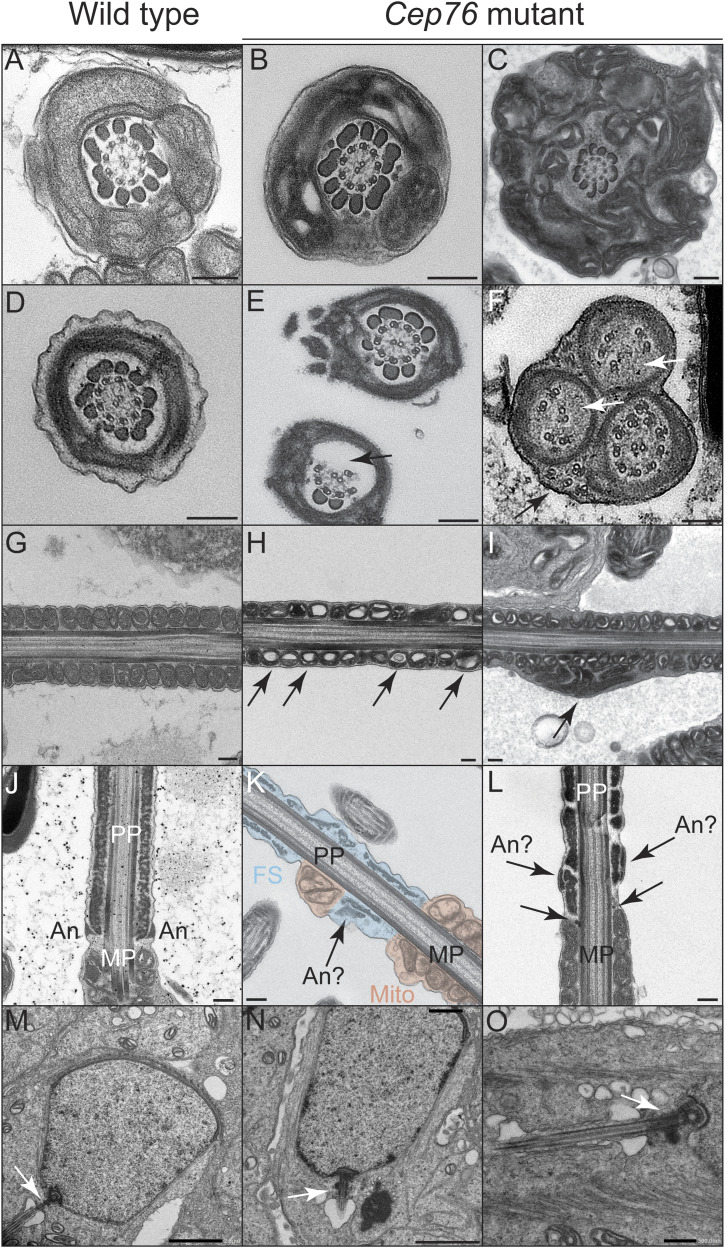
CEP76 is required for accessory structure assembly and normal axoneme ultrastructure. **(A, B, C, D, E, F, G, H, I, J, K, L)** Wild-type sperm are shown in panels (A, D, G, J); sperm from *Cep76* mutants are shown in panels (B, C, E, F, H, I, K, L). Top row (A, B, C)—sperm midpiece cross-sections highlighted a broadly normal axoneme in both genotypes, with evidence of mitochondrial and membrane aggregation in mutant sperm. Second row (D, E, F)—sperm principal piece cross-sections highlighted the absence of some microtubule doublets and outer dense fibres (arrow, (E)), and the displacement of some microtubule doublets (black and white arrows, (F)) in mutant cells. Third row (G, H, I)—midpiece longitudinal sections highlighted abnormal mitochondrial morphology (arrows, (H)) and aggregation (arrow, (I)) in mutant cells. Fourth row (J, K, L)—longitudinal sections of the midpiece–principal piece boundary revealed abnormal annulus formation (arrows point to predicted annulus structures, (K, L)) and the consequential mixing of mitochondria and fibrous sheath structures in mutant cells (K). Bottom row (M, N, O)—no major differences were apparent in the transition zone and early axoneme development in early elongating spermatids between genotypes. MP, midpiece; PP, principal piece; An, annulus; FS, fibrous sheath; Mito, mitochondria. Scale bars = 200 nm, except for (M, N) where scale bars = 2 μm, and (O) where the scale bar = 500 nm.

**Figure 4.**
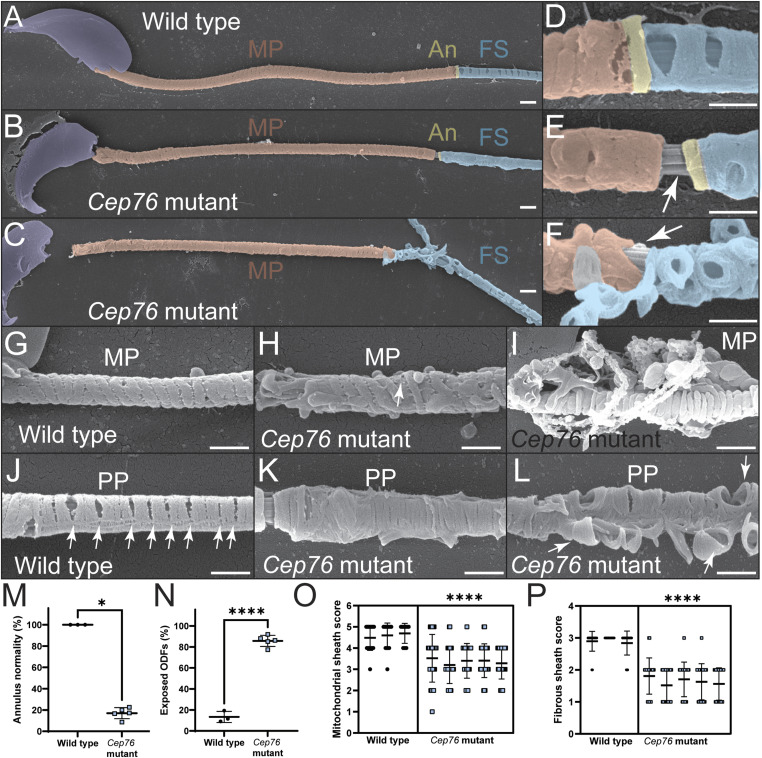
Annulus, mitochondrial sheath, and fibrous sheath formation are impaired in the absence of CEP76. **(A, B, C)** Scanning electron microscopy on wild-type (A) and mutant (B, C) membrane-stripped sperm revealed defects in accessory structures in mutant sperm. MP, midpiece; An, annulus; FS, fibrous sheath. Sperm nuclei are overlaid in purple, mitochondria in red, annuli in yellow, and fibrous sheath in blue. **(D, E, F)** High-power images of the midpiece–principal piece boundary (D, E, F) revealed an abnormal annulus region in mutant sperm and the core axoneme being exposed (arrows). **(G, H, I)** Wild-type mitochondria were helically arranged with normal morphology (G), whereas mitochondria were poorly arranged (H) or aggregated (I) in mutant cells. **(J)** In addition, the slits present in wild-type sperm fibrous sheath ((J), arrows) were missing in mutant cells. **(C, K, L)** Fibrous sheath deposition was highly abnormal in the absence of CEP76 (C, K, L), and circumferential ribs appeared to be unanchored in mutant cells. Scale bars = 1 μm. **(M, N, O, P)** These defects were quantified (M, N, O, P) using the following: **(M)** annulus normality assessment, **(N)** prevalence of exposed outer dense fibres, **(O)** mitochondrial sheath normality assessment (5 = best, 1 = worst), and **(P)** fibrous sheath normality assessment (3 = best, 1 = worst). *P < 0.05, ****P < 0.0001. All n = 3–5.

**Figure S4.**
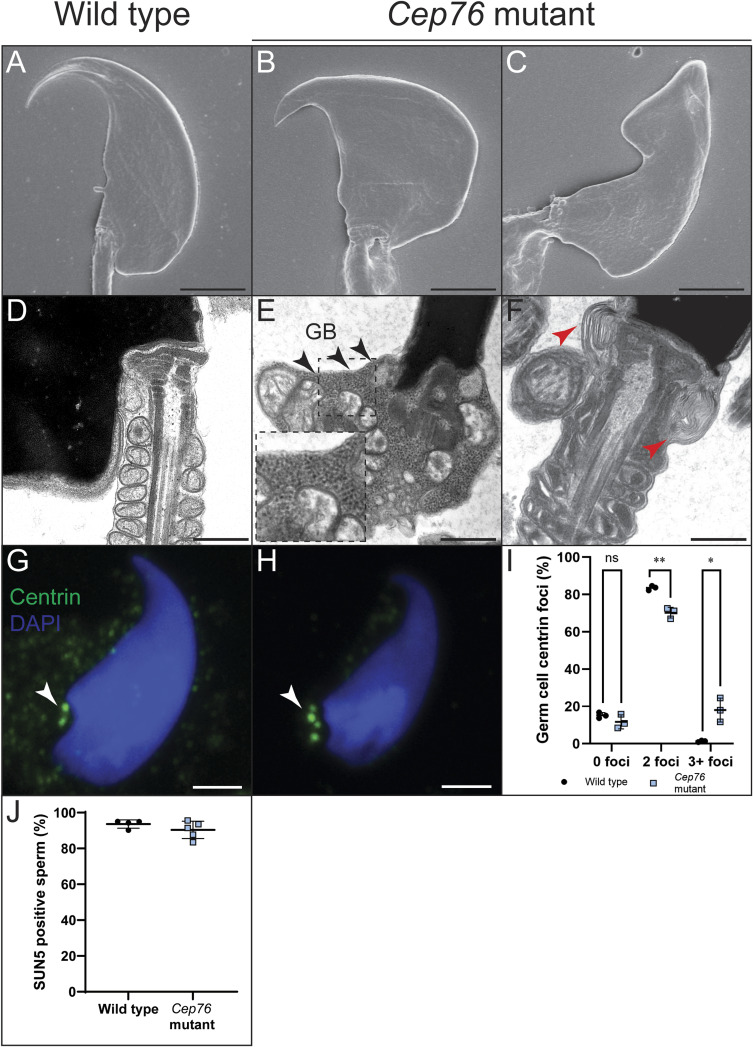
CEP76 is required for sperm neck integrity and suppression of centriole duplication in male germ cells. **(A, B, C)** Scanning electron microscopy images of sperm heads from wild-type and *Cep76* mutant sperm. **(D, E, F)** Transmission electron microscopy images of sperm neck regions. **(E)** Arrows and expanded dashed boxes denote potential granulated bodies throughout the neck region in panel (E). In panel (F), the red arrows point to abnormal membrane folding. **(G, H)** Centriole number/content was defined by centrin staining. Arrows point to individual centriole components. Scale bars = 2 μm in all panels, except length in (D, E, F) where they are 1 μm. **(I)** Quantification of the centriole number in round and elongating spermatids, categorised as 0, 2, or 3+ foci (n = 3). **(J)** Incidence of sperm SUN5 staining. *P < 0.05, **P < 0.01, ns, not significant.

To define the origins of the motility and structural defects, we investigated sperm ultrastructure using transmission electron microscopy (TEM) and scanning electron microscopy (SEM) on isolated epididymal sperm (Figs 3 and 4 and S4). At the midpiece level, transverse TEM sections revealed that axoneme ultrastructure appeared superficially normal, bearing all outer dense fibres and the 9 + 2 microtubule formation with dynein arms in sperm from wild-type and mutant males (Fig 3A and B, respectively). In *Cep76* mutant sperm, however, a build-up of mitochondria and membranes was seen (Fig 3C). Longitudinal TEM images of sperm tail sections of the midpiece revealed an intact mitochondrial sheath in wild type (Fig 3G). In sperm from mutant males, abnormal mitochondria with enlarged spacing within mitochondrial matrixes were observed (Fig 3H), including notable mitochondrial aggregation (Fig 3I—arrow).

TEM investigation of the axoneme ultrastructure at the principal piece level revealed absent outer dense fibres and microtubule doublets in many sperm tails from mutant males (Fig 3D–F). In addition, we observed the abnormal formation of the annulus. Specifically, in sperm from *Cep76* mutant males the annulus was not clearly distinguishable (Fig 3K), or not identifiable (Fig 3L). SEM of sperm from *Cep76* mutant males further emphasised the poorly formed annulus and accessory structures in the midpiece and principal piece (Fig 4). In sperm from wild-type males, the annulus was positioned at the junction between the mitochondrial sheath of the midpiece and the fibrous sheath of the principal piece (Fig 4A and D). In sperm from *Cep76* mutant males, although the annulus was often associated with the fibrous sheath, it was rarely found aligned at the distal end of the mitochondrial sheath (Fig 4B, C, E, and F). As a result, a region of exposed ODFs was evident between the mitochondrial sheath and the annulus or fibrous sheath. Quantification of the defects in annulus positioning (Fig 4M) revealed normal positioning and structure of the annulus in only 10% of sperm from mutant males compared with 95% of sperm from wild-type males (*P* = 0.036). Consequently, we quantified the incidence of ODF-exposed regions at the distal midpiece (Fig 4N). This revealed that ODFs were exposed in 97% of sperm from *Cep76* mutants but only in 10% of sperm from wild type (*P* < 0.0001). In *Cep76* mutants, the annulus was often found embedded within the proximal region of the fibrous sheath (e.g., Fig 4K) rather than at the junction of the midpiece, or it was not clearly observed because of large deformations in the fibrous sheath (Fig 4F). Because of the predicted role of CEP76 in TZ function, we anticipate this is due to a defect in TZ/annulus migration rather than the fibrous sheath assembling too far proximally. This phenotype, along with the ectopic localisation of SEPT4 as shown in Fig 2G, suggests that CEP76 plays a role in establishing the formation/structural competency and movement of the annulus.

In addition, we observed rare examples of mitochondria ectopically incorporated within the principal piece in *Cep76* knockout sperm (Fig 3K), but never in sperm from wild-type controls, that is, the presence of overlapping fibrous sheath and mitochondria. Mitochondrial packing in epididymal sperm from *Cep76* mutants was irregular and characterised by misaligned and poorly compacted mitochondria along the mitochondrial sheath (Fig 4H versus Fig 4G). Mitochondrial aggregation defects identified via TEM were confirmed via SEM and were characterised by collections of poorly formed mitochondria and a build-up around the midpiece (Fig 4I). Scoring of mitochondrial sheath normality (as defined in the Materials and Methods section; Fig 4O) revealed a significant reduction in mitochondrial sheath quality in sperm from *Cep76* mutant males compared with wild type (3.4 in mutant versus 4.6 in wild type; *P* < 0.0001).

Moreover, although in wild-type sperm, the fibrous sheath appeared intact and continuous via TEM (Fig 3J), in mutant sperm, we observed fragmented fibrous sheath components (Fig 3K and L). SEM confirmed that fibrous sheath formation was notably impaired in the absence of CEP76 (Fig 4C, F, K, and L). In wild-type sperm, the fibrous sheath is comprised of two longitudinal columns linked by circumferential ribs. Gaps in the fibrous sheath were evident at regular intervals (Fig 4D and J). In the absence of CEP76, however, gaps/slits between circumferential ribs were rare (Fig 4K), and in many cases, the ribs appeared to be unanchored and projected from the body of the tail (Fig 4L). Notably, the proximal portion of the fibrous sheath appeared to be the most disorganised (e.g., Fig 4C). Fibrous sheath normality was scored (as detailed in the Materials and Methods section; Fig 4P), which revealed a highly significant reduction in fibrous sheath quality in sperm from *Cep76* mutants (1.6 in mutant versus 2.9 in wild type; *P* < 0.0001).

Examination of the sperm neck region by TEM (Fig S4D–F) revealed a number of abnormal structures, including imperfect capitulum structures and a build-up of mitochondria and ectopic vesicles in sperm from *Cep76* mutant males. Furthermore, an excess of what we predicted to be granulated bodies was seen throughout the cytoplasm (Fig S4E). Granulated bodies are transported into the ciliary compartment to form the outer dense fibres, which is continuous with the HTCA (43). This finding strongly supports a role of CEP76 in the selective entry of proteins and vesicles into the ciliary compartment during spermiogenesis and, by extension, in building of the ODF and the fortification of the HTCA. Of relevance, we did not observe a difference in the proportion of sperm positive for SUN5 staining between genotypes (Fig S4J; *P* = 0.41). SUN5 is a protein required to fortify the connection between the capitulum and the basal plate (44), that is, the junction between the tail and the nucleus. As this structure is proximal to where the TZ exists, these data are consistent with a role of CEP76 in the TZ and the assembly of more distal regions of the tail, and that the capitulum defects are secondary effects of CEP76 loss.

To assess whether the defects described above were associated with structural abnormalities in the TZ as sperm tails are forming, we undertook TEM on early elongating spermatids. As shown in Fig 3M–O, TZ structures were comparable between genotypes.

Collectively, these data reveal CEP76 is a key determinant in the development of multiple aspects of sperm tail development, including the fibrous sheath, annulus positioning, and the mitochondrial sheath.

### Loss of CEP76 leads to aberrant sperm composition

To explore the hypothesis that CEP76 is involved in the selective entry of proteins into the ciliary lobe, we performed quantitative mass spectrometry on sperm from the cauda epididymis of *Cep76* mutant and wild-type males (Table 1A). Here, we identified 63 differentially expressed proteins in sperm from mutant males (35 up-regulated and 28 down-regulated), including multiple mitochondrial and apoptotic proteins. This difference in mitochondrial protein content is consistent with the abnormal accumulation of mitochondria around the sperm tail as detailed above. In agreement with the observed reduced tail length, alpha-tubulin content was significantly reduced in sperm from *Cep76* mutant males. In a separate analysis, and to account for differences in tail length, the content of known (or predicted) sperm tail proteins was normalised to alpha-tubulin. As shown in Table 1B, after this normalisation, 12 proteins were identified as significantly altered in sperm from *Cep76* mutant males compared with wild type including several that are essential for sperm tail function and male fertility. We also identified 23 proteins that were only present in a single genotype (i.e., wild-type or mutant sperm). No direct sperm motility–regulating proteins of interest were identified in this last cohort. Candidates of interest to explain sperm dysfunction included the following: axonemal protein DNAH2 (1.65-fold wild-type levels, *P* = 0.041), the actin-based motor protein myosin 9 (3.5-fold wild-type levels, *P* = 0.017), and AKAP3 and AKAP4, which are major components of the fibrous sheath (24) (1.36-fold and 1.46-fold wild-type levels, *P* = 0.032 and *P* = 0.05, respectively).

**Table 1.** (A) Proteins with significantly altered content in sperm from *Cep76* mutants. (B) Proteins with significantly altered content in sperm from *Cep76* mutants, after normalisation to alpha-tubulin content.

A. Protein full name	Short	WT1	WT2	WT3	MT1	MT2	MT3	*P*-value	Content	Panther protein class
*Proteins present in only one genotype (23 proteins—18 up-regulated* and *5 down-regulated)*										
Lactotransferrin	LTF	Not detected			13	14	14	<0.0001	Up	Transfer/carrier protein
Tubulin beta-6 chain	TUBB6	Not detected			10	12	10	0.0001	Up	Tubulin
Inactive ribonuclease-like protein 10	RNASE10	Not detected			1	2	1	0.016	Up	Endoribonuclease
Myosin-14	MYH14	Not detected			2	5	3	0.019	Up	Actin-binding cytoskeletal protein
Cysteine-rich secretory protein 3	CRISP3	Not detected			2	2	2	NA	Up	Defence/immunity protein
Deoxyribonuclease-1-like 2	DNASE1L2	Not detected			1	3	2	0.026	Up	Endodeoxyribonuclease
Glia-derived nexin	SERPINE2	Not detected			3	2	1	0.026	Up	Protease inhibitor
Sperm-associated antigen 11	SPAG11	Not detected			1	1	1	NA	Up	—
Lymphocyte antigen 6 complex locus protein G5c	LY6G5C	Not detected			1	1	1	NA	Up	—
Eppin	EPPIN	Not detected			2	2	2	NA	Up	—
Solute carrier family 25 member 35	SLC25A35	Not detected			1	1	1	NA	Up	—
Surfeit locus protein 1	SURF1	Not detected			1	1	1	NA	Up	Chaperone
Septin-8	SEPTIN8	Not detected			1	1	1	NA	Up	Cytoskeletal protein
NADH dehydrogenase 1 alpha subcomplex assembly factor 3	NDUFAF3	Not detected			1	1	1	NA	Up	—
Testican-1	SPOCK1	Not detected			1	1	1	NA	Up	Extracellular matrix glycoprotein
Probable leucine--tRNA ligase, mitochondrial	LARS2	Not detected			1	1	1	NA	Up	Aminoacyl-tRNA synthetase
Beta-defensin 40	DEFB40	Not detected			1	1	1	NA	Up	Antimicrobial response protein
Serine protease inhibitor Kazal-type 11	SPINK11	Not detected			1	1	1	NA	Up	Protease inhibitor
BH3-interacting domain death agonist S	BID	1	1	2	Not detected			0.016	Down	—
Solute carrier family 22 member 16	SLC22A16	1	1	1	Not detected			NA	Down	Secondary carrier transporter
Nucleoporin NDC1	NDC1	1	1	1	Not detected			NA	Down	—
Protein archease	ZBTB8OS	1	1	1	Not detected			NA	Down	—
Phosphoglucomutase-like protein 5	PGM5	1	1	1	Not detected			NA	Down	Mutase
*Proteins present in both genotypes but at different levels (63 proteins—35 up-regulated* and *28 down-regulated)*										
Calcium-activated chloride channel regulator 3A-1	CLCA3A1	1	2	0	18	19	16	0.0001	Up	Ion channel
Dipeptidyl peptidase 3	DPP3	12	12	12	7	5	6	0.0005	Down	Metalloprotease
Heterogeneous nuclear ribonucleoprotein K	HNRNPK	1	1	0	6	8	6	0.0013	Up	RNA metabolism protein
Peptidyl-prolyl cis–trans isomerase F, mitochondrial	PPIF	1	1	0	4	3	3	0.0048	Up	—
UV excision repair protein RAD23 homolog B	RAD23B	5	5	6	3	3	2	0.0048	Down	Damaged DNA-binding protein
Matrilysin	MMP7	1	1	0	10	11	6	0.027	Up	Metalloprotease
Puromycin-sensitive aminopeptidase	NPEPPS	4	5	6	2	2	1	0.0075	Down	Metalloprotease
Leucine-rich repeat-containing protein 1	LRRC1	2	3	2	4	4	4	0.0075	Up	—
Protein phosphatase 1B	PPM1B	12	12	12	10	11	10	0.0075	Down	Protein phosphatase
Prostaglandin-H2 D-isomerase	PTGDS	1	2	1	3	4	4	0.0078	Up	Transfer/carrier protein
Epididymal-specific lipocalin-5	LCN5	1	0	0	3	3	5	0.011	Up	Transfer/carrier protein
Tubulin alpha-8	TUBA8	19	19	20	17	18	17	0.013	Down	Tubulin
3-Ketodihydrosphingosine reductase	KDSR	0	1	0	3	2	2	0.013	Up	Reductase
Gamma-glutamylcyclotransferase	GGCT	3	2	3	1	1	0	0.013	Down	—
Binder of sperm protein homolog 2	BSPH2	1	1	0	2	3	3	0.013	Up	—
Bifunctional coenzyme A synthase	COASY	2	3	3	1	1	0	0.013	Down	Kinase
Mitochondrial amidoxime reducing component 2	MARC2	2	3	2	4	5	4	0.013	Up	—
Glutathione peroxidase 3	GPX3	3	3	0	6	6	6	0.016	Up	Peroxidase
Histidine triad nucleotide-binding protein 2, mitochondrial	HINT2	2	3	3	4	4	4	0.016	Up	Nucleotide phosphatase
Succinyl-CoA:3-ketoacid coenzyme A transferase 1, mitochondrial	OXCT1	4	4	3	5	5	5	0.016	Up	Transferase
MICOS complex subunit Mic27	APOOL	2	2	1	3	3	3	0.016	Up	—
MICOS complex subunit MIC13	MICOS13	1	1	0	2	2	2	0.016	Up	—
Beta-defensin 15	DEFB15	1	1	0	2	2	2	0.016	Up	—
Apoptosis-inducing factor 1	AIFM1	1	2	2	3	3	3	0.016	Up	Oxidoreductase
Hormone-sensitive lipase	LIPE	4	4	4	2	3	3	0.016	Down	Lipase
BPI fold-containing family A member 3	BPIFA3	3	3	3	2	1	2	0.016	Down	—
Single-stranded DNA-binding protein, mitochondrial	SSBP1	1	0	1	2	2	2	0.016	Up	DNA metabolism protein
Proteasome subunit beta type-2	PSMB2	5	6	5	4	4	4	0.016	Up	Protease
Proteasomal ubiquitin receptor ADRM1	ADRM1	2	2	2	1	0	0	0.016	Down	—
Phosphatidylinositol transfer protein alpha isoform	PITPNA	2	3	4	1	0	0	0.016	Down	Transporter
Cytochrome c, testis-specific	CYCT	4	5	4	3	3	3	0.016	Down	—
Myosin-9	MYH9	3	15	6	23	34	25	0.017	Up	Actin-binding cytoskeletal protein
Alpha-1-antitrypsin 1-6	SERPINA1F	2	3	0	6	5	5	0.018	Up	Protease inhibitor
Keratin, type II cytoskeletal 5	KRT5	3	0	0	5	7	5	0.018	Up	—
Keratin, type II cytoskeletal 2 epidermal	KRT2	1	1	0	3	2	2	0.024	Up	—
Nucleoporin NUP35	NUP35	2	3	2	1	1	0	0.024	Down	Transporter
Acylpyruvase FAHD1, mitochondrial	FAHD1	3	2	2	1	0	1	0.024	Down	Hydrolase
Proteasome subunit beta type-6	PSMB6	5	6	5	3	4	4	0.024	Down	Protease
Presequence protease, mitochondrial	PITRM1	4	5	4	3	2	3	0.024	Down	Metalloprotease
DnaJ homolog subfamily B member 4	DNAJB4	2	1	0	3	3	4	0.025	Up	Chaperone
NPC intracellular cholesterol transporter	NPC2	1	1	0	3	2	4	0.025	Up	—
Protein phosphatase inhibitor 2	PPP1R2	6	6	7	5	4	3	0.025	Down	Phosphatase inhibitor
Ubiquitin carboxyl-terminal hydrolase isozyme L5	UCHL5	6	6	7	3	5	4	0.025	Down	Cysteine protease
ATP synthase subunit alpha, mitochondrial	ATP5F1A	21	23	21	24	24	24	0.025	Up	ATP synthase
Leukotriene A-4 hydrolase	LTA4H	10	7	12	5	3	0	0.027	Down	—
Serine protease HTRA1	HTRA1	3	2	0	4	5	5	0.034	Up	Serine protease
Prolyl endopeptidase	PREP	5	4	7	3	2	1	0.034	Down	Serine protease
NADP-dependent malic enzyme	ME1	14	11	14	10	10	8	0.038	Down	Oxidoreductase
Carboxypeptidase M	CPM	2	2	0	3	5	4	0.039	Up	Protease
Ester hydrolase C11orf54 homolog	—	4	4	6	3	2	1	0.039	Down	—
BAG family molecular chaperone regulator 1 Bag1	BAG1	6	4	4	3	2	1	0.039	Down	Chaperone
Succinate dehydrogenase [ubiquinone] iron–sulphur subunit, mitochondrial	SDHB	11	13	10	14	16	14	0.039	Up	Dehydrogenase
Keratin, type I cytoskeletal 42	KRT42	0	3	0	4	4	4	0.040	Up	Intermediate filament
26S proteasome regulatory subunit 4	PSMC1	12	16	14	11	8	10	0.041	Down	Protease
Dynein heavy chain 2, axonemal	DNAH2	4	8	8	12	11	10	0.041	Up	Microtubule-binding motor protein
Carnitine O-palmitoyltransferase 2, mitochondrial	CPT2	15	18	19	13	13	8	0.043	Down	Acyltransferase
Cytochrome c1, haem protein, mitochondrial	CYC1	8	8	7	7	6	6	0.047	Down	—
Probable inactive ribonuclease-like protein 13	RNASE13	1	0	0	1	2	2	0.047	Up	Endoribonuclease
Glutathione S-transferase theta-2	GSTT2	1	2	2	1	0	0	0.047	Down	Transferase
GPI-anchor transamidase	PIGK	0	0	1	1	2	2	0.047	Up	—
Retinol dehydrogenase 14	RDH14	1	2	2	0	1	0	0.047	Down	Dehydrogenase
Adipocyte plasma membrane–associated protein	APMAP	1	4	3	5	6	5	0.047	Up	—
Myosin light polypeptide 6	MYL6	0	2	3	4	5	4	0.049	Up	Actin-binding cytoskeletal protein
**B. Protein full name**	**Short**	**WT1**	**WT2**	**WT3**	**MT1**	**MT2**	**MT3**	***P***-**value**	**Specific to sperm tail?**	**PANTHER protein class**
Tubulin beta-5 chain	TUBB5	1.11	1.21	1.1	1.35	1.28	1.41	0.017	**N**	Tubulin
Calnexin	CANX	0.47	0.58	0.7	0.88	1.11	0.82	0.032	**N**	Chaperone
A-kinase anchor protein 3	AKAP3	1.53	1.74	1.25	1.88	2.22	2.06	0.033	**Y**	Scaffold/adapter protein
60S acidic ribosomal protein P0	RPLP0	0.21	0.26	0.3	0.35	0.33	0.41	0.037	**N**	Ribosomal protein
MYCBP-associated protein	MYCBPAP	0.16	0.16	0.05	0.24	0.22	0.29	0.038	**N**	—
Heat shock 70 kD protein 1-like	HSPA1L	1.42	1.21	1.2	1.59	1.5	1.47	0.039	**N**	Hsp70 family chaperon
Carboxylesterase 1D	CES1D	0.37	0.37	0.2	0.71	0.44	0.65	0.042	**N**	Esterase
Kelch-like protein 10	KLHL10	0.32	0.21	0.25	0.47	0.33	0.41	0.043	**N**	Scaffold/adapter protein
Integral membrane protein 2B	ITM2B	0.16	0.11	0.05	0.29	0.33	0.18	0.044	**N**	—
Ras-related protein Rab-5C	RAB5C	0.21	0.26	0.25	0.29	0.28	0.29	0.047	**N**	Small GTPase
UMP-CMP kinase	CMPK1	0.26	0.21	0.2	0.35	0.28	0.29	0.050	**N**	Nucleotide kinase
A-kinase anchor protein 4	AKAP4	2.84	3	1.7	3.53	3.78	3.71	0.050	**Y**	Scaffold/adapter protein

(A) Green proteins are up-regulated in sperm from mutant mice, and blue proteins are down-regulated. (B) All proteins were up-regulated in sperm from the mutant. Known sperm tail proteins are highlighted in orange. Other proteins are present throughout the entire sperm cell, or localisation is unknown. WT, wild type; MT, mutant. NA = not applicable because of identical spectral counts in both samples; thus, *t* test cannot be used.

**Figure 5.**
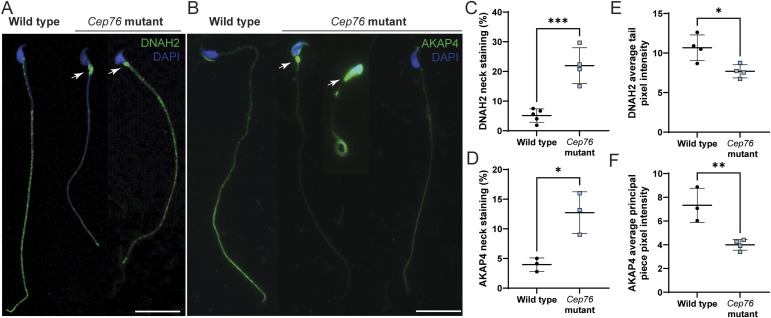
CEP76 is required for the loading of essential motility and fibrous sheath proteins into the sperm tail. **(A)** Wild-type versus *Cep76* mutant data for (A) DNAH2 localisation in cauda epididymal sperm and **(B)** AKAP4 localisation in cauda epididymal sperm. Scale bars = 20 μm. Arrows point to the accumulation of DNAH2 or AKAP4 in the neck region of sperm. **(C, D)** Number of sperm with this neck localisation was quantified, as shown in (C, D), for DNAH2 and AKAP4, respectively. **(E, F)** Average tail pixel intensity (per area) of DNAH2 and AKAP4 was quantified and is shown in (E, F). ***P < 0.001, **P < 0.01, *P < 0.05. All n = 3–5.

To investigate this counterintuitive increase in several motility proteins, we defined the localisation of DNAH2 and AKAP4 proteins in sperm from *Cep76* mutant males using immunofluorescence (Fig 5). As expected, in wild-type sperm, the dynein arm protein DNAH2 was localised throughout the midpiece and principal piece of the tail (Fig 5A) (45, 46). In contrast, although DNAH2 was found within the sperm tail in many sperm from *Cep76* mutants, it was notably accumulated in the neck region. In other sperm, DNAH2 was seen nearly exclusively localised to the neck and appeared to be almost absent from the tail (Fig 5A). Specifically, 22% of sperm from *Cep76* mutant males displayed an accumulation of DNAH2 in the neck region compared with 5.1% of sperm from wild types (4.3 x wild-type levels, *P* = 0.0078; Fig 5C). Tail pixel intensity analysis (per area) revealed a 29% reduction in DNAH2 content across the entire flagellum of sperm from mutant mice compared with wild-type controls (*P* = 0.0040; Fig 5E).

Also as expected for a component of the fibrous sheath, AKAP4 was primarily localised to the principal piece sperm from wild type (Fig 5B). As above, in *Cep76* mutant sperm, AKAP4 accumulated in the neck region in 13% of sperm compared with 4% of sperm from wild-type mice (Fig 5D). Quantification of neck AKAP4 staining revealed 3.1-fold levels in *Cep76* mutant sperm relative to wild type (*P* = 0.040; Fig 5D). An analysis of pixel intensity revealed a significant, 47% reduction in AKAP4 content (per area) within the principal piece of sperm from *Cep76* mutant males compared with wild type (*P* < 0.0001; Fig 5F).

Collectively, these data underscore an essential role of CEP76 in TZ function and the regulated entry of key sperm tail proteins into the ciliary compartment and sperm tail development.

### CEP76 is not required for manchette formation and migration

SEM confirmed the magnitude of head deformation in sperm from *Cep76* mutant males (Fig S4A–C). The manchette is critically involved in shaping the distal half of the sperm nucleus and further acts as a transport freeway for protein delivery to the basal body and into the sperm tail (26). Despite the highly irregular sperm head shape, the formation of the manchette appeared normal in the absence of CEP76 as identified by alpha-tubulin staining of isolated germ cells (Fig S3F) and testis sections (Fig S3G). Electron microscopy reinforced that the manchette structure was overtly normal (Fig S3H), including a normal perinuclear ring and manchette microtubules. This analysis does not, however, exclude the possibility of subtle defects in manchette structure and/or kinetics.

### CEP76 is required for the maintenance of centriole number in male germ cells

CEP76 has previously been linked to a role in the suppression of centriole duplication in 293T cells (39), thus raising the possibility of a similar role in germ cells. To explore this hypothesis, we stained purified round and elongating spermatids with the centriole marker centrin (Fig S4G and H) and quantified the number of centrioles per cell (Fig S4I). As expected, two distinct centrin foci were observed in 84% of wild-type spermatids and only 1% of wild-type cells exhibited 3+ centrin foci. In contrast, in spermatids from *Cep76* mutant males, 18% of cells possessed 3 or more centriole structures (*P* = 0.046) and only 70% of cells contained 2 centrin foci (*P* = 0.0076). These data confirm a role of CEP76 in germ cell centriole duplication suppression. Despite this finding, sperm from *Cep76* mutants contained only single tails and axonemes; that is, centriole overduplication did not lead to multiple basal bodies and axoneme growth.

## Discussion

Building a sperm tail is a complex and multistep process, requiring the coordinated action of multiple protein and organelle transport processes (reviewed in references 26, 47), and is absolutely essential for male fertility. One centrosome is inherited per spermatid during the process of meiosis. It subsequently duplicates, but does not separate, and matures to give rise to the basal body that docks to both the plasma and nuclear membranes (48, 49). From this structure, the sperm tail, a modified cilium, forms within a discrete, sealed ciliary compartment. To enter the ciliary compartment, all proteins must pass through the TZ that sits at the junction between the cytoplasm and the ciliary compartment. Assuming that cilium formation during germ cell development is analogous to primary cilia wherein TZ function has been studied, all component proteins for the axoneme, outer dense fibres, and fibrous sheath must be selectively transported through the TZ. In this study, we establish CEP76 as what we predict is the first known protein to play a male germ cell–specific role in the TZ and demonstrate that CEP76 is required for the development of functionally competent sperm. We show CEP76 facilitates the incorporation of tubulin into the tail and that an absence of CEP76 leads to short sperm tails. We also show CEP76 is essential for the efficient transport of AKAP4 protein into the flagellum compartment, underpinning normal fibrous sheath development. Equally, it optimises the entry of DNAH2, a core component of the axoneme motility apparatus. In the absence of CEP76, the sperm axoneme is functionally incompetent. CEP76 is also required for the integrity and appropriate migration positioning of the annulus and thus likely influences mitochondrial sheath length. In addition to its TZ function, our data reveal that analogous to its roles in somatic cells (39), CEP76 plays a role in the suppression of centriole duplication in haploid male germ cells. This study highlights *CEP76* as a bona fide male fertility gene in men and mice and adds to the growing evidence that several genetic factors contribute to both sperm morphology and sperm count.

The following discussion is premised on the idea that CEP76 functions in the TZ. It has recently been suggested that during all steps of sperm tail development before annulus migration, the annulus and TZ are part of the same ciliary structure (31). Our data support this idea. A number of core components of the TZ, as established in cilia (e.g., CEP290, MKS1), are also present in the annulus, and several of these proteins remain localised to the annulus during its migration in late spermiogenesis (17). Consistent with this, the abnormal annulus structures and the fragmented SEPT4 localisation pattern observed in sperm from *Cep76* mutant males suggest that TZ structure influences annulus structure and function. Specifically, we show that CEP76 is required for annulus positioning, and in its absence, the annulus is poorly formed and migrates a shorter distance. Very little is known about factors that govern annulus migration and positioning (50), but these processes do not appear to be influenced by the development of the fibrous sheath, which ultimately borders the annulus in mature spermatids but is formed before annulus migration (22, 32, 51). Equally, the absence of an annulus in *Sept4* and *Sept12* mutant models did not impair fibrous sheath formation (30, 34). In the current study, we identified proteins ectopically localised at the sperm neck region. We hypothesise that this accumulation occurred before annulus migration when the TZ is still functional, that is, concordant with the assembly of the axoneme, ODF, and fibrous sheath (see Graphical Abstract). After the migration of the annulus and the presumed destruction of the classic TZ structure, just before spermiation, any protein accumulated at the sperm neck would be free to move some way into the midpiece. Future studies are required to test this hypothesis.

It has been hypothesised that within CEP76, the C2 and transglutaminase domains cooperate to allow CEP76 to play a role in the TZ as a Y-shaped linker (42). Y-shaped linker structures form the main body of the TZ; proteins of which can be grouped into the Meckel syndrome (MKS) and nephronophthisis (NPHP) complexes (52). These complexes participate in multiple roles during ciliogenesis—TZ attachment, core axoneme extension, and then regulation of components’ entry into the cilia/flagella (53). Based on domain architecture and functional predictions, CEP76 has been hypothesised to span both MKS and NPHP complexes (42). The presence of a C2 domain in CEP76 strongly suggests it anchors at the side of the Y-shaped linker interacting with the plasma membrane (42). Our data highlight that, in vivo, CEP76 is not essential for axoneme formation, for example, compared with CEP290, where CEP290 absence precludes TZ formation and ciliogenesis altogether (54, 55). Rather, data suggest that CEP76 plays a facilitative role in TZ function specifically in male germ cells and is likely not a core Y-linker component. As mice did not show symptoms of MKS or NPHP, our data reveal CEP76 is not essential for somatic cell TZ function. The possibility exists, however, that mice possessed subtle defects in somatic tissues.

Furthermore, our proteomics results revealed the content of only a relatively small number of sperm tail proteins was altered in the absence of CEP76. We accept, however, that additional proteins may have been equally mislocalised, but as their overall content per sperm was unaltered, they were therefore overlooked in our mass spectrometry analysis. As such, AKAP4 and DNAH2 should be considered a minimum list of CEP76 target proteins, supporting the hypothesis that CEP76 is highly selective in its function and that other TZ proteins are required to form a functioning sperm tail. The absence of pathology in somatic tissues is, however, consistent with the concept that TZ content is cell type–specific and that different TZ proteins are involved in the selective recruitment of precise subsets of proteins from the many thousands of proteins present within the cell cytoplasm; that is, the TZ functions as a cell type–specific filter controlling protein entry into cilia. As a specific example in support of the hypothesis that CEP76 is required for full TZ function, we identified a reduction in alpha-tubulin protein content in sperm from *Cep76* mutants, consistent with the reduction in sperm tail length. Although a reduction in sperm tail length could otherwise be explained by disruption to the intraflagellar transport pathway (56), the reduced protein content within the body of the tail, the presence of ectopic protein localisation to the sperm neck (external to the TZ), and prior data implicating CEP76 in TZ function (42) strongly suggest an essential role of CEP76 in male germ cell TZ function. Indeed, data from the ciliopathy field (reviewed in references 16, 57) highlight that loss-of-function mutations across different TZ proteins induce a broad spectrum of pathologies that partly overlap, but are distinct depending on the affected gene. This suggests individual TZ genes/proteins serve tissue-specific functions.

Overt consequences of CEP76 loss included the abnormal assembly and content of sperm tail axoneme and accessory structures. We observed inappropriate packaging of the fibrous sheath and the absence of spacing between the circumferential ribs. AKAP4 is the most abundant fibrous sheath protein, constituting nearly half its entire content (58), and acts as a scaffold protein for both the longitudinal columns and ribs, whereas AKAP3 plays a similar role in the ribs of the fibrous sheath (23, 59). The fibrous sheath defects seen in the absence of CEP76 are consistent with the poor entry of major fibrous sheath proteins into the sperm tail compartment via the TZ and abnormal fibrous sheath compaction. Similarly, we identified an accumulation of granulated bodies in the sperm neck region and the partial absence of outer dense fibres in the principal piece axoneme. Collectively, our results strongly suggest the deficit in the entry of fibrous sheath and outer dense fibre components into the tail compartment resulted in compromised accessory structure development and thus directly impaired sperm motility.

Finally, we identified significant aggregation of mitochondria and morphology in the midpiece. The mitochondrial sheath is the last accessory structure to be loaded onto the sperm tail. This occurs contemporaneously with the migration of the annulus and plasma membrane down to a position immediately proximal to, and abutting, the principal piece, as marked by the start of the fibrous sheath (26, 60). Although membrane migration occurs at the same time as annulus migration, it does not appear to be driven by the annulus as the former occurs even when the annulus does not form (30, 32). Although the annulus is strictly not required for mitochondrial sheath formation, in its absence, mitochondrial morphology is frequently abnormal (30, 33, 61). Before their loading, spherical mitochondria are recruited from the cytoplasm and ordered in four columns parallel to the axoneme. They then move towards the core of the tail and attach to the outer dense fibres, elongate, coil, and stagger to intercalate around the midpiece (60). Next, mitochondria elongate and attach end-to-end to form a double helix around the axoneme. *Cep76* mutant sperm mitochondria were largely elongated and uniform along the midpiece, suggesting that mitochondrial recruitment and early mitochondrial elongation processes proceeded normally. We predict the shorter annulus migration distance in sperm from *Cep76* mutant males leaves insufficient space for the normal number of mitochondria to coil around the sperm tail, that is, to sterically interfere with the later processes of mitochondrial elongation and prevent their tight compaction within the midpiece (62). Although unlikely, CEP76 may also be involved directly in recruiting mitochondria into the midpiece. Alternate hypotheses to explain abnormal mitochondrial morphology and enlarged crista spacing include a reduced ability of the annulus to act as a diffusion barrier (potentially adding additional cellular stress via osmoregulation); or that the sperm are beginning to undergo activation of the apoptotic pathways, noting that the mitochondria are the origin of a truncated cell death pathway in sperm (63).

Our data revealed a second function of CEP76 in male germ cells—a role in suppressing supernumerary centrioles, analogous to its role identified in human sarcoma cells in vitro (39). A previously established mechanism highlighted that CEP76 controls centriole duplication via interaction with PLK1 and CP110 (38, 39). Although not investigated directly here, we note *Cep76* mutant mice were viable and were free of any overt body–wide disease, suggesting that CEP76 is not essential for centriole function in somatic tissues. Furthermore, the absence of double axonemes and tails suggests these centriole components are non-functional. A similar role in regulating centriole number in male germ cells (64) has been identified for another TZ protein, DZIP1 (65). The finding that two predicted TZ proteins, CEP76 and DZIP1, influence centriole number, reiterates the functional relationship between the centriole and the TZ (see Graphical Abstract). Specifically, the TZ is a structure formed from, and just distal to, the distal centriole that sits at the interface between the sperm head and the tail.

To the best of our knowledge, this is the first example of a protein with a predicted germ cell–specific function in the TZ. In the absence of CEP76, essential components of the sperm tail are unable to enter the ciliary lobe, meaning less or minimal incorporation into the growing tail and thus male infertility. These data provide support for the concept that TZ composition is cell type–specific and that the TZ provides an additional layer of specificity to the composition and function of cilia and flagella.

## Materials and Methods

### Ethics statement

Experimental procedures involving mice followed animal ethics guidelines generated by the Australian National Health and Medical Research Council (NHMRC). All animal experiments were approved by the Animal Experimentation Ethics Committee (BSCI/2017/31) at Monash University, or The University of Melbourne Animal Ethics Committee (application 20640). Mice were housed on a 14-h light cycle with temperature set to 20°C and standard environmental enrichment.

### Mutant mouse production

As described previously (36), exome sequencing of infertile men identified *CEP76* as a high-confidence candidate male fertility gene. The patient carried a homozygous c.607G>C (p.Gly203Arg) missense variant in a conserved residue of *CEP76*, which was predicted to be intolerant to variation (Fig S1). Pathogenicity assessments using SIFT and PolyPhen-2 (66, 67) predicted the missense variant to affect function (SIFT) and to be possibly damaging (PolyPhen). To test the requirement for CEP76 in male fertility, *Cep76* loss-of-function mice were generated on the C57BL/6J background through the Monash University Genome Modification Platform (a partner of the Australian Phenomics Network) using the CRISPR/Cas9 technology. Excision of exon 3 of *Cep76* was undertaken with CRISPR guide sequences targeting regions flanking exon 3: upstream—TTTAAAACTCAGTTCGTGGT; and downstream—GGTCTACATAGTAAAGTTCT. This was predicted to lead to a premature stop codon in exon 4 and a truncated protein in the only full protein-coding transcript of *Cep76* (ENSMUST00000097542.3) but did not target the smaller second transcript encoding exons 5-12. Changes in the gene sequence were identified with Sanger sequencing (Supplemental Data 1). Mice heterozygous for the *Cep76* deletion were intercrossed to generate mutant individuals and wild-type controls. Genotyping was performed by Transnetyx. A reduction in the *Cep76* truncated transcript level was investigated using qRT–PCR on testis cDNA (primers F—GCGGCTCGATTTGTTAATGT; and R—AGTCCCCACACAGACAAAGG) relative to *Ppia* (primers F—GTCTCCTTCGAGCTGTTT; and R—ACCCTGGACATGAATCCT). Similarly, confirmation that all transcripts contained the *Cep76* deletion was done using primers in the deleted region (F—CCCTTCTTCTCCAAAGCAAACCG; and R—CGAGCAAATCTGTCCAGGCAA).

Supplemental Data 1. *Cep76* mutant model validation.

### CEP76 species alignments

CEP76 protein alignments were conducted using the protein Basic Local Alignment Search Tool (NCBI). Sequences used were *Homo sapiens* ENSP00000262127; *Pan troglodytes* ENSPTRP00000016812; *Macaca mulatta* ENSMMUP00000069818; *Rattus norvegicus* ENSRNOP00000034590; *Mus musculus* ENSMUSP00000095149; *Danio rerio* ENSDARP00000075595. We compared entire protein identity across species and focused on the conservation of amino acid 203G, which was mutated in the infertile patient (Fig S1B).

### Analysis of *Cep76* expression

Whole organ RNA was extracted from adult mouse brain, epididymis, heart, liver, lung, spleen, and testis, to investigate the expression of *Cep76* across different tissues. Each tissue was homogenised in TRIzol Reagent to isolate RNA, which was converted to cDNA using 50 μM oligo(dt)15 primers with SuperScript III enzyme, and used for qRT–PCR with SYBR Green Master Mix as previously described (68). Primers used to detect *Cep76* were F—CTCGGTCACCAGCAATGAAA; and R—CAGACAGTGGTGAGGCCAAG, and the housekeeping gene *Ppia* is denoted above. We also used published RNA-seq data for human organs to assess *CEP76* expression (40) and single-cell RNA-sequencing data contained within FertilityOnline (mcg.ustc.edu.cn/bsc/spermgenes2.0/index.html) to investigate *Cep76* expression in mouse testes (69).

### Fertility analysis

Mutant males (*Cep76*^−/−^) and wild-type male littermates (*Cep76*^*+/+*^) were aged 10–14 wk, and their fertility was assessed using the pipeline outlined previously (70). In brief, five mice of each genotype were set up to mate with two independent wild-type females each (6–12 wk old). The presence of a copulatory plug was recorded as an indication of successful mating. Litter sizes were recorded as the number of pups generated per plug. Males were subsequently culled and weighed (10–14 wk of age), and one testis and epididymis were dissected and processed for histological assessment. Additional testes and epididymides were snap-frozen on dry ice for calculation of daily sperm production and epididymal sperm counts as described previously (71). In brief, testes were weighed and resuspended in a detergent-based solution and sonicated to burst the nuclei of cells with non-compacted DNA while leaving resistant elongated spermatids and sperm nuclei intact. Detergent-resistant nuclei were counted on a haemocytometer (71), and the total number of cells within the testis was calculated. To calculate daily sperm production, this number was divided by 4.84—the length of time in days step 14–16 spermatids (detergent-resistant) spent in the testis (72). To measure the total epididymal sperm count, the same method was used without the calculation to obtain a daily count. Before incubation in the detergent solution, the epididymides were finely minced.

In addition, sperm were collected from the cauda of the epididymis through backflushing, then resuspended in MT6 medium at 37°C for motility assessment via computer-assisted semen analysis (73). Residual sperm were washed in PBS, then dried onto SuperFrost slides overnight. Sperm were then fixed in 4% PFA for 10 min, washed in PBS, and stained with Mayer’s haematoxylin for 10 min and eosin (Amber Scientific) for 1 min to allow an assessment of sperm morphology and tail length. Alternatively, fixed sperm were permeabilised in 0.1% Triton X-100/PBS (Sigma-Aldrich) for 10 min, washed in PBS, and stained with 10 μg/ml DAPI (Thermo Fisher Scientific) to allow an assessment of sperm head morphology, or 1 μg/ml peanut agglutinin conjugated to Alexa Fluor 488 to label the acrosome. Head morphology assessment was performed using Nuclear Morphology Analysis software version 1.17.1 (74) via an ImageJ plugin (National Institutes of Health, USA). All length measurements (midpiece length, principal piece length, full tail length, and annulus positioning) were measured using ImageJ (version 1.52k).

### Electron microscopy

To investigate germ cell ultrastructure, testes were processed for electron microscopy as outlined previously (75). Similarly, caudal sperm were backflushed into MT6 medium and processed for electron microscopy as outlined previously (75). Images were taken either on a Jeol 1400 Plus electron microscope at the Vera and Clive Ramaciotti Centre for Electron Microscopy (Monash University, Australia), or on a Talos L120C or a FEI Teneo VolumeScope at the Ian Holmes Imaging Center (The University of Melbourne). To view the mitochondria, annulus, and fibrous sheath structure of sperm via SEM, sperm were isolated from the cauda epididymis and incubated in 100 μl of 1× PBS for 30 min to strip the plasma membrane, then processed as outlined in reference (76).

### Scoring of mitochondrial sheath, fibrous sheath, and annulus normality

To quantify the degree of ultrastructural defects within sperm tail structures, scoring of each structure (mitochondrial sheath, fibrous sheath, and annulus) was performed on SEM images. For each biological replicate, at least 25 sperm were assessed. Mitochondrial sheath normality was scored (1–5): 1—missing mitochondria; 2—many abnormally oriented and/or thick mitochondria; 3—few abnormally oriented and/or thick mitochondria; (4)—broadly normal with very few abnormally oriented mitochondria; and (5)—no defects, where “normal” was defined as homogeneously coiled mitochondria, with none missing, thick, or abnormally oriented. Fibrous sheath normality was scored from ranks (1–3): 1—no slits and/or massive aggregation of circumferential ribs or longitudinal columns; 2—reduced number of slits or slight aggregation; and 3—normal structure, where normal was defined as slits occurring at regular intervals and no aggregation. Annulus normality was scored as follows: normal—intact, not shrunken, and localised to the junction between the midpiece and the principal piece; or abnormal—small, ectopically placed, or unidentifiable.

### Mass spectrometry to define sperm protein composition

Sperm were backflushed from cauda epididymides of adult wild-type (one mouse per replicate, *n* = 3 replicates) and *Cep76* mutant (two mice per replicate because of reduction in epididymal sperm content) males into MT6 medium for 15 min at 37°C. Sperm were washed three times in 1× Tris-buffered saline, pelleted, and then stored at −80°C. Sperm pellets were then dried in a speed vacuum, prepared for liquid chromatography–tandem mass spectrometry, and run on a SCIEX QTRAP6500 as described previously (77).

Data were assessed as (1) total/raw spectral counts or (2) total spectral counts were normalised to the alpha-tubulin content (tubulin alpha-8 chain) for a comparison of sperm tail protein content in recognition of the shorter sperm tails measured from *Cep76* null mice. Sperm tail proteins were identified via proteomics or localisation studies. For completeness, proteins with an unknown localisation or that are localised throughout the head and tail were included in the tail protein group. Proteins associated exclusively with the sperm head or neck and mitochondrial proteins were not included here as the normalisation process (to alpha-tubulin) as they would not be influenced by processes involved in selective entry into the ciliary compartment. A two-tailed *t* test was used to determine significant differences in protein content between genotypes. All data are available in Table S1. As we have not corrected for multiple comparisons in our proteomics data and have used zero values for proteins not detected in individual biological replicates, the biological significance of proteins identified via this screen as being significantly modulated in mutant sperm should be interpreted with caution.

Table S1 Proteomics raw spectral counts.

### Immunofluorescence and protein localisation

To define centriole number and manchette structure in male germ cells, spermatids were isolated from the testes of males using the STAPUT method (78). Cells were settled onto poly-L-lysine–coated SuperFrost slides for 15 min and then fixed in ice-cold methanol at −20°C for a maximum of 7 min. Slides were immediately washed and rehydrated in PBS. Fixed elongating spermatids were permeabilised in 0.2% Triton X-100/PBS for 10 min, blocked in CAS-Block (Dako), incubated overnight in 0.1 μg/ml centrin antibody (04-1624; Merck) to stain centriole components and beta-tubulin (ab21057; Abcam) to stain the manchette for spermatid staging, then counterstained with DAPI. For manchette imaging, spermatids were incubated overnight with an alpha-tubulin antibody (T5168, 1:1,000; Sigma-Aldrich), then stained with secondary goat anti-mouse 488 (Abcam) and DAPI. Z-stacks of labelled cells were taken using a Leica SP8 confocal and flattened to capture all slices in a single frame. The number of centriole components per spermatid was then counted for 100 cells per replicate, per genotype.

To define the localisation of a subset of differentially expressed proteins, fixed sperm were permeabilised in 0.2% Triton X-100/PBS, blocked in CAS-Block, incubated overnight in primary antibodies (0.5 μg/ml DNAH2 [64309; Invitrogen], 2.5 μg/ml SEPT4 [166788; Abcam]) at 4°C, stained with relevant fluorescent secondary antibodies (Thermo Fisher Scientific) for 1 h, then counterstained with DAPI. For other antibodies (2.8 μg/ml AKAP4 [24986-1-AP; ProteinTech], 0.8 μg/ml SUN5 [17495-1-AP; ProteinTech]), sperm were permeabilised in 0.5% Triton X-100/PBS. For the assessment of sperm mitochondrial sheath length, live sperm were loaded with 5 μM MitoTracker Red CMXRos in MT6 solution for 30 min as per the manufacturer’s instructions. Sperm were then washed in PBS, fixed in 4% PFA, and allowed to settle onto slides, as described above. All images were taken using cellSens software (Olympus) on an Olympus BX-53 microscope (Olympus) equipped with an Olympus 392 DP80 camera or a Leica SP8 confocal. Ectopic localisation of DNAH2 and AKAP4 was scored in at least 100 sperm per biological replicate. Immunofluorescence staining intensity was used as a surrogate of protein content in sperm tails. FIJI v2.1.0 was used to trace sperm tails (freehand line tool) and measure the average pixel intensity for AKAP4 and DNAH2 staining in at least 20 sperm, to account for differences in tail length.

CEP76 localisation in testis sections, germ cells, and sperm was attempted with three antibodies: ab86613; Abcam, #A302-326A; Bethyl Laboratories, and HPA039395; Sigma-Aldrich (Human Protein Atlas). All antibodies reacted with antigens in mutant tissue, suggesting they bound non-specifically. Equally, all antibodies bound non-specifically to non-CEP76 proteins when used in Western blotting (data not shown).

### Statistical analysis

Statistical analyses to determine the significance between wild-type and *Cep76* mutant data were performed in Prism 9 (GraphPad). For all data that were normally distributed with sufficient sample size (Shapiro–Wilk test), we used a *t* test with Welch’s correction to account for variance. For data not normally distributed, we used a Mann–Whitney *U* test. A *P*-value less than 0.05 was considered significant. No data were excluded.

## Supplementary Material

Reviewer comments

## Data Availability

Mass spectrometry data are available in Table S1.

## References

[1] Inaba K (2011) Sperm flagella: Comparative and phylogenetic perspectives of protein components. Mol Hum Reprod 17: 524–538. 10.1093/molehr/gar034 21586547

[2] Garcia-Gonzalo FR, Corbit KC, Sirerol-Piquer MS, Ramaswami G, Otto EA, Noriega TR, Seol AD, Robinson JF, Bennett CL, Josifova DJ, (2011) A transition zone complex regulates mammalian ciliogenesis and ciliary membrane composition. Nat Genet 43: 776–784. 10.1038/ng.891 21725307 PMC3145011

[3] Hu Q, Milenkovic L, Jin H, Scott MP, Nachury MV, Spiliotis ET, Nelson WJ (2010) A septin diffusion barrier at the base of the primary cilium maintains ciliary membrane protein distribution. Science 329: 436–439. 10.1126/science.1191054 20558667 PMC3092790

[4] Chih B, Liu P, Chinn Y, Chalouni C, Komuves LG, Hass PE, Sandoval W, Peterson AS (2011) A ciliopathy complex at the transition zone protects the cilia as a privileged membrane domain. Nat Cell Biol 14: 61–72. 10.1038/ncb2410 22179047

[5] Goncalves J, Pelletier L (2017) The ciliary transition zone: Finding the pieces and assembling the gate. Mol Cell 40: 243–253. 10.14348/molcells.2017.0054 PMC5424270 28401750

[6] Nachury MV, Seeley ES, Jin H (2010) Trafficking to the ciliary membrane: How to get across the periciliary diffusion barrier? Annu Rev Cell Dev Biol 26: 59–87. 10.1146/annurev.cellbio.042308.113337 19575670 PMC2952038

[7] Arima T, Shibata Y, Yamamoto T (1984) A deep-etching study of the Guinea pig tracheal cilium with special reference to the ciliary transitional region. J Ultrastruct Res 89: 34–41. 10.1016/s0022-5320(84)80021-0 6544881

[8] Reiter JF, Blacque OE, Leroux MR (2012) The base of the cilium: Roles for transition fibres and the transition zone in ciliary formation, maintenance and compartmentalization. EMBO Rep 13: 608–618. 10.1038/embor.2012.73 22653444 PMC3388784

[9] Dean S, Moreira-Leite F, Varga V, Gull K (2016) Cilium transition zone proteome reveals compartmentalization and differential dynamics of ciliopathy complexes. Proc Natl Acad Sci U S A 113: E5135–E5143. 10.1073/pnas.1604258113 27519801 PMC5024643

[10] Gorden NT, Arts HH, Parisi MA, Coene KL, Letteboer SJ, van Beersum SE, Mans DA, Hikida A, Eckert M, Knutzen D, (2008) CC2D2A is mutated in Joubert syndrome and interacts with the ciliopathy-associated basal body protein CEP290. Am J Hum Genet 83: 559–571. 10.1016/j.ajhg.2008.10.002 18950740 PMC2668034

[11] Li C, Jensen VL, Park K, Kennedy J, Garcia-Gonzalo FR, Romani M, De Mori R, Bruel AL, Gaillard D, Doray B, (2016) MKS5 and CEP290 dependent assembly pathway of the ciliary transition zone. PLoS Biol 14: e1002416. 10.1371/journal.pbio.1002416 26982032 PMC4794247

[12] Lambacher NJ, Bruel AL, van Dam TJ, Szymanska K, Slaats GG, Kuhns S, McManus GJ, Kennedy JE, Gaff K, Wu KM, (2016) TMEM107 recruits ciliopathy proteins to subdomains of the ciliary transition zone and causes Joubert syndrome. Nat Cell Biol 18: 122–131. 10.1038/ncb3273 26595381 PMC5580800

[13] Avidor-Reiss T, Leroux MR (2015) Shared and distinct mechanisms of compartmentalized and cytosolic ciliogenesis. Curr Biol 25: R1143–R1150. 10.1016/j.cub.2015.11.001 26654377 PMC5857621

[14] Klena N, Pigino G (2022) Structural biology of cilia and intraflagellar transport. Annu Rev Cell Dev Biol 38: 103–123. 10.1146/annurev-cellbio-120219-034238 35767872

[15] Wei Q, Xu Q, Zhang Y, Li Y, Zhang Q, Hu Z, Harris PC, Torres VE, Ling K, Hu J (2013) Transition fibre protein FBF1 is required for the ciliary entry of assembled intraflagellar transport complexes. Nat Commun 4: 2750. 10.1038/ncomms3750 24231678 PMC3856926

[16] Wang L, Wen X, Wang Z, Lin Z, Li C, Zhou H, Yu H, Li Y, Cheng Y, Chen Y, (2022) Ciliary transition zone proteins coordinate ciliary protein composition and ectosome shedding. Nat Commun 13: 3997. 10.1038/s41467-022-31751-0 35810181 PMC9271036

[17] Basiri ML, Ha A, Chadha A, Clark NM, Polyanovsky A, Cook B, Avidor-Reiss T (2014) A migrating ciliary gate compartmentalizes the site of axoneme assembly in Drosophila spermatids. Curr Biol 24: 2622–2631. 10.1016/j.cub.2014.09.047 25447994 PMC4254545

[18] Vieillard J, Paschaki M, Duteyrat JL, Augiere C, Cortier E, Lapart JA, Thomas J, Durand B (2016) Transition zone assembly and its contribution to axoneme formation in Drosophila male germ cells. J Cell Biol 214: 875–889. 10.1083/jcb.201603086 27646273 PMC5037411

[19] Reiter JF, Leroux MR (2017) Genes and molecular pathways underpinning ciliopathies. Nat Rev Mol Cell Biol 18: 533–547. 10.1038/nrm.2017.60 28698599 PMC5851292

[20] Woolley D (2000) The molecular motors of cilia and eukaryotic flagella. Essays Biochem 35: 103–115. 10.1042/bse0350103 12471893

[21] Leung MR, Roelofs MC, Ravi RT, Maitan P, Henning H, Zhang M, Bromfield EG, Howes SC, Gadella BM, Bloomfield-Gadêlha H, (2021) The multi-scale architecture of mammalian sperm flagella and implications for ciliary motility. EMBO J 40: e107410. 10.15252/embj.2020107410 33694216 PMC8013824

[22] Miki K, Willis WD, Brown PR, Goulding EH, Fulcher KD, Eddy EM (2002) Targeted disruption of the Akap4 gene causes defects in sperm flagellum and motility. Dev Biol 248: 331–342. 10.1006/dbio.2002.0728 12167408

[23] Eddy EM, Toshimori K, O’Brien DA (2003) Fibrous sheath of mammalian spermatozoa. Microsc Res Tech 61: 103–115. 10.1002/jemt.10320 12672126

[24] Brown PR, Miki K, Harper DB, Eddy EM (2003) A-kinase anchoring protein 4 binding proteins in the fibrous sheath of the sperm flagellum. Biol Reprod 68: 2241–2248. 10.1095/biolreprod.102.013466 12606363

[25] Miyata H, Oura S, Morohoshi A, Shimada K, Mashiko D, Oyama Y, Kaneda Y, Matsumura T, Abbasi F, Ikawa M (2021) SPATA33 localizes calcineurin to the mitochondria and regulates sperm motility in mice. Proc Natl Acad Sci U S A 118: e2106673118. 10.1073/pnas.2106673118 34446558 PMC8536318

[26] Pleuger C, Lehti MS, Dunleavy JE, Fietz D, O’Bryan MK (2020) Haploid male germ cells-the Grand Central Station of protein transport. Hum Reprod Update 26: 474–500. 10.1093/humupd/dmaa004 32318721

[27] Gu NH, Zhao WL, Wang GS, Sun F (2019) Comparative analysis of mammalian sperm ultrastructure reveals relationships between sperm morphology, mitochondrial functions and motility. Reprod Biol Endocrinol 17: 66. 10.1186/s12958-019-0510-y 31416446 PMC6696699

[28] Ricci M, Breed WG (2005) Morphogenesis of the fibrous sheath in the marsupial spermatozoon. J Anat 207: 155–164. 10.1111/j.1469-7580.2005.00437.x 16050902 PMC1571513

[29] Fawcett DW (1975) The mammalian spermatozoon. Dev Biol 44: 394–436. 10.1016/0012-1606(75)90411-x 805734

[30] Kissel H, Georgescu MM, Larisch S, Manova K, Hunnicutt GR, Steller H (2005) The Sept4 septin locus is required for sperm terminal differentiation in mice. Dev Cell 8: 353–364. 10.1016/j.devcel.2005.01.021 15737931

[31] Avidor-Reiss T, Ha A, Basiri ML (2017) Transition zone migration: A mechanism for cytoplasmic ciliogenesis and postaxonemal centriole elongation. Cold Spring Harb Perspect Biol 9: a028142. 10.1101/cshperspect.a028142 28108487 PMC5538411

[32] Kwitny S, Klaus AV, Hunnicutt GR (2010) The annulus of the mouse sperm tail is required to establish a membrane diffusion barrier that is engaged during the late steps of spermiogenesis. Biol Reprod 82: 669–678. 10.1095/biolreprod.109.079566 20042538 PMC2842486

[33] Kuo YC, Lin YH, Chen HI, Wang YY, Chiou YW, Lin HH, Pan HA, Wu CM, Su SM, Hsu CC, (2012) SEPT12 mutations cause male infertility with defective sperm annulus. Hum Mutat 33: 710–719. 10.1002/humu.22028 22275165

[34] Shen YR, Wang HY, Kuo YC, Shih SC, Hsu CH, Chen YR, Wu SR, Wang CY, Kuo PL (2017) SEPT12 phosphorylation results in loss of the septin ring/sperm annulus, defective sperm motility and poor male fertility. PLoS Genet 13: e1006631. 10.1371/journal.pgen.1006631 28346465 PMC5386304

[35] Ho HC, Wey S (2007) Three dimensional rendering of the mitochondrial sheath morphogenesis during mouse spermiogenesis. Microsc Res Tech 70: 719–723. 10.1002/jemt.20457 17457821

[36] Nagirnaja L, Lopes AM, Charng WL, Miller B, Stakaitis R, Golubickaite I, Stendahl A, Luan T, Friedrich C, Mahyari E, (2022) Diverse monogenic subforms of human spermatogenic failure. Nat Commun 13: 7953. 10.1038/s41467-022-35661-z 36572685 PMC9792524

[37] Houston BJ, Riera-Escamilla A, Wyrwoll MJ, Salas-Huetos A, Xavier MJ, Nagirnaja L, Friedrich C, Conrad DF, Aston KI, Krausz C, (2021) A systematic review of the validated monogenic causes of human male infertility: 2020 update and a discussion of emerging gene-disease relationships. Hum Reprod Update 28: 15–29. 10.1093/humupd/dmab030 34498060 PMC8730311

[38] Barbelanne M, Chiu A, Qian J, Tsang WY (2016) Opposing post-translational modifications regulate Cep76 function to suppress centriole amplification. Oncogene 35: 5377–5387. 10.1038/onc.2016.74 27065328 PMC5125818

[39] Tsang WY, Spektor A, Vijayakumar S, Bista BR, Li J, Sanchez I, Duensing S, Dynlacht BD (2009) Cep76, a centrosomal protein that specifically restrains centriole reduplication. Dev Cell 16: 649–660. 10.1016/j.devcel.2009.03.004 19460342 PMC4062978

[40] Fagerberg L, Hallstrom BM, Oksvold P, Kampf C, Djureinovic D, Odeberg J, Habuka M, Tahmasebpoor S, Danielsson A, Edlund K, (2014) Analysis of the human tissue-specific expression by genome-wide integration of transcriptomics and antibody-based proteomics. Mol Cell Proteomics 13: 397–406. 10.1074/mcp.M113.035600 24309898 PMC3916642

[41] Avidor-Reiss T, Fishman EL (2019) It takes two (centrioles) to tango. Reproduction 157: R33–R51. 10.1530/REP-18-0350 30496124 PMC6494718

[42] Zhang D, Aravind L (2012) Novel transglutaminase-like peptidase and C2 domains elucidate the structure, biogenesis and evolution of the ciliary compartment. Cell Cycle 11: 3861–3875. 10.4161/cc.22068 22983010 PMC3495828

[43] Yang K, Meinhardt A, Zhang B, Grzmil P, Adham IM, Hoyer-Fender S (2012) The small heat shock protein ODF1/HSPB10 is essential for tight linkage of sperm head to tail and male fertility in mice. Mol Cell Biol 32: 216–225. 10.1128/MCB.06158-11 22037768 PMC3255718

[44] Shang Y, Zhu F, Wang L, Ouyang YC, Dong MZ, Liu C, Zhao H, Cui X, Ma D, Zhang Z, (2017) Essential role for SUN5 in anchoring sperm head to the tail. Elife 6: e28199. 10.7554/eLife.28199 28945193 PMC5634783

[45] Hwang JY, Nawaz S, Choi J, Wang H, Hussain S, Nawaz M, Lopez-Giraldez F, Jeong K, Dong W, Oh JN, (2021) Genetic defects in DNAH2 underlie male infertility with multiple morphological abnormalities of the sperm flagella in humans and mice. Front Cell Dev Biol 9: 662903. 10.3389/fcell.2021.662903 33968937 PMC8103034

[46] Li Y, Sha Y, Wang X, Ding L, Liu W, Ji Z, Mei L, Huang X, Lin S, Kong S, (2019) DNAH2 is a novel candidate gene associated with multiple morphological abnormalities of the sperm flagella. Clin Genet 95: 590–600. 10.1111/cge.13525 30811583

[47] Lehti MS, Sironen A (2016) Formation and function of the manchette and flagellum during spermatogenesis. Reproduction 151: R43–R54. 10.1530/REP-15-0310 26792866

[48] Larsson M, Norrander J, Graslund S, Brundell E, Linck R, Stahl S, Höög C (2000) The spatial and temporal expression of Tekt1, a mouse tektin C homologue, during spermatogenesis suggest that it is involved in the development of the sperm tail basal body and axoneme. Eur J Cell Biol 79: 718–725. 10.1078/0171-9335-00097 11089920

[49] Avasthi P, Scheel JF, Ying G, Frederick JM, Baehr W, Wolfrum U (2013) Germline deletion of Cetn1 causes infertility in male mice. J Cell Sci 126: 3204–3213. 10.1242/jcs.128587 23641067 PMC3711207

[50] Guan J, Kinoshita M, Yuan L (2009) Spatiotemporal association of DNAJB13 with the annulus during mouse sperm flagellum development. BMC Dev Biol 9: 23. 10.1186/1471-213X-9-23 19298648 PMC2670831

[51] Xu K, Yang L, Zhang L, Qi H (2020) Lack of AKAP3 disrupts integrity of the subcellular structure and proteome of mouse sperm and causes male sterility. Development 147: dev181057. 10.1242/dev.181057 31969357

[52] Williams CL, Li C, Kida K, Inglis PN, Mohan S, Semenec L, Bialas NJ, Stupay RM, Chen N, Blacque OE, (2011) MKS and NPHP modules cooperate to establish basal body/transition zone membrane associations and ciliary gate function during ciliogenesis. J Cell Biol 192: 1023–1041. 10.1083/jcb.201012116 21422230 PMC3063147

[53] Garcia-Gonzalo FR, Reiter JF (2017) Open sesame: How transition fibers and the transition zone control ciliary composition. Cold Spring Harb Perspect Biol 9: a028134. 10.1101/cshperspect.a028134 27770015 PMC5287074

[54] Wu Z, Pang N, Zhang Y, Chen H, Peng Y, Fu J, Wei Q (2020) CEP290 is essential for the initiation of ciliary transition zone assembly. PLoS Biol 18: e3001034. 10.1371/journal.pbio.3001034 33370260 PMC7793253

[55] Rachel RA, Yamamoto EA, Dewanjee MK, May-Simera HL, Sergeev YV, Hackett AN, Pohida K, Munasinghe J, Gotoh N, Wickstead B, (2015) CEP290 alleles in mice disrupt tissue-specific cilia biogenesis and recapitulate features of syndromic ciliopathies. Hum Mol Genet 24: 3775–3791. 10.1093/hmg/ddv123 25859007 PMC4459394

[56] San Agustin JT, Pazour GJ, Witman GB (2015) Intraflagellar transport is essential for mammalian spermiogenesis but is absent in mature sperm. Mol Biol Cell 26: 4358–4372. 10.1091/mbc.E15-08-0578 26424803 PMC4666132

[57] Hildebrandt F, Benzing T, Katsanis N (2011) Ciliopathies. N Engl J Med 364: 1533–1543. 10.1056/NEJMra1010172 21506742 PMC3640822

[58] Eddy EM, O’Brien DA, Fenderson BA, Welch JE (1991) Intermediate filament--like proteins in the fibrous sheath of the mouse sperm flagellum. Ann N Y Acad Sci 637: 224–239. 10.1111/j.1749-6632.1991.tb27312.x 1723852

[59] Mandal A, Naaby-Hansen S, Wolkowicz MJ, Klotz K, Shetty J, Retief JD, Coonrod SA, Kinter M, Sherman N, Cesar F, (1999) FSP95, a testis-specific 95-kilodalton fibrous sheath antigen that undergoes tyrosine phosphorylation in capacitated human spermatozoa. Biol Reprod 61: 1184–1197. 10.1095/biolreprod61.5.1184 10529264

[60] Otani H, Tanaka O, Kasai K, Yoshioka T (1988) Development of mitochondrial helical sheath in the middle piece of the mouse spermatid tail: Regular dispositions and synchronized changes. Anat Rec 222: 26–33. 10.1002/ar.1092220106 3189885

[61] Ihara M, Kinoshita A, Yamada S, Tanaka H, Tanigaki A, Kitano A, Goto M, Okubo K, Nishiyama H, Ogawa O, (2005) Cortical organization by the septin cytoskeleton is essential for structural and mechanical integrity of mammalian spermatozoa. Dev Cell 8: 343–352. 10.1016/j.devcel.2004.12.005 15737930

[62] Shimada K, Park S, Miyata H, Yu Z, Morohoshi A, Oura S, Matzuk MM, Ikawa M (2021) ARMC12 regulates spatiotemporal mitochondrial dynamics during spermiogenesis and is required for male fertility. Proc Natl Acad Sci U S A 118: e2018355118. 10.1073/pnas.2018355118 33536340 PMC8017931

[63] Aitken RJ, Whiting S, De Iuliis GN, McClymont S, Mitchell LA, Baker MA (2012) Electrophilic aldehydes generated by sperm metabolism activate mitochondrial reactive oxygen species generation and apoptosis by targeting succinate dehydrogenase. J Biol Chem 287: 33048–33060. 10.1074/jbc.M112.366690 22851170 PMC3463304

[64] Lv M, Liu W, Chi W, Ni X, Wang J, Cheng H, Li WY, Yang S, Wu H, Zhang J, (2020) Homozygous mutations in DZIP1 can induce asthenoteratospermia with severe MMAF. J Med Genet 57: 445–453. 10.1136/jmedgenet-2019-106479 32051257 PMC7361034

[65] Lapart JA, Gottardo M, Cortier E, Duteyrat JL, Augiere C, Mange A, Jerber J, Solassol J, Gopalakrishnan J, Thomas J, (2019) Dzip1 and Fam92 form a ciliary transition zone complex with cell type specific roles in Drosophila. Elife 8: e49307. 10.7554/eLife.49307 31821146 PMC6904220

[66] Ng PC, Henikoff S (2003) SIFT: Predicting amino acid changes that affect protein function. Nucleic Acids Res 31: 3812–3814. 10.1093/nar/gkg509 12824425 PMC168916

[67] Adzhubei IA, Schmidt S, Peshkin L, Ramensky VE, Gerasimova A, Bork P, Kondrashov AS, Sunyaev SR (2010) A method and server for predicting damaging missense mutations. Nat Methods 7: 248–249. 10.1038/nmeth0410-248 20354512 PMC2855889

[68] Houston BJ, Nagirnaja L, Merriner DJ, O’Connor AE, Okuda H, Omurtag K, Smith C, Aston KI, Conrad DF, O’Bryan MK (2021) The Sertoli cell expressed gene secernin-1 (Scrn1) is dispensable for male fertility in the mouse. Dev Dyn 250: 922–931. 10.1002/dvdy.299 33442887 PMC9012169

[69] Ernst C, Eling N, Martinez-Jimenez CP, Marioni JC, Odom DT (2019) Staged developmental mapping and X chromosome transcriptional dynamics during mouse spermatogenesis. Nat Commun 10: 1251. 10.1038/s41467-019-09182-1 30890697 PMC6424977

[70] Houston BJ, Conrad DF, O’Bryan MK (2021) A framework for high-resolution phenotyping of candidate male infertility mutants: From human to mouse. Hum Genet 140: 155–182. 10.1007/s00439-020-02159-x 32248361 PMC9014372

[71] Dunleavy JEM, O’Connor AE, Okuda H, Merriner DJ, O’Bryan MK (2021) KATNB1 is a master regulator of multiple katanin enzymes in male meiosis and haploid germ cell development. Development 148: dev199922. 10.1242/dev.199922 34822718

[72] Oakberg EF (1956) Duration of spermatogenesis in the mouse and timing of stages of the cycle of the seminiferous epithelium. Am J Anat 99: 507–516. 10.1002/aja.1000990307 13402729

[73] Gibbs GM, Orta G, Reddy T, Koppers AJ, Martinez-Lopez P, de la Vega-Beltran JL, Lo JC, Veldhuis N, Jamsai D, McIntyre P, (2011) Cysteine-rich secretory protein 4 is an inhibitor of transient receptor potential M8 with a role in establishing sperm function. Proc Natl Acad Sci U S A 108: 7034–7039. 10.1073/pnas.1015935108 21482758 PMC3084142

[74] Skinner BM, Rathje CC, Bacon J, Johnson EEP, Larson EL, Kopania EEK, Good JM, Yousafzai G, Affara NA, Ellis PJI (2019) A high-throughput method for unbiased quantitation and categorization of nuclear morphology†. Biol Reprod 100: 1250–1260. 10.1093/biolre/ioz013 30753283 PMC6497523

[75] Dunleavy JEM, Okuda H, O’Connor AE, Merriner DJ, O’Donnell L, Jamsai D, Bergmann M, O’Bryan MK (2017) Katanin-like 2 (KATNAL2) functions in multiple aspects of haploid male germ cell development in the mouse. PLoS Genet 13: e1007078. 10.1371/journal.pgen.1007078 29136647 PMC5705150

[76] Korneev D, Merriner DJ, Gervinskas G, de Marco A, O’Bryan MK (2021) New insights into sperm ultrastructure through enhanced scanning electron microscopy. Front Cell Dev Biol 9: 672592. 10.3389/fcell.2021.672592 33968944 PMC8100687

[77] Netherton J, Hetherington L, Ogle R, Gavgani M, Velkov T, Villaverde A, Tanphaichitr N, Baker MA (2020) Mass spectrometry reveals new insights into the production of superoxide anions and 4-hydroxynonenal adducted proteins in human sperm. Proteomics 20: e2070024. 10.1002/pmic.201900205 31846556

[78] Dunleavy JEM, O’Connor AE, O’Bryan MK (2019) An optimised STAPUT method for the purification of mouse spermatocyte and spermatid populations. Mol Hum Reprod 25: 675–683. 10.1093/molehr/gaz056 31642475

