# Supplementary material for: Genetic mutation of Cep76 results in male infertility due to abnormal sperm tail composition
Source: Life Sci Alliance. 2024 Apr 3;7(6):e202302452. doi: 10.26508/lsa.202302452 (PMC10992998; doi:10.26508/lsa.202302452)
Supplement: Supplementary file 1 [file LSA-2023-02452_Supplemental_Data_1.docx]

**Wild type sequence** (blue = exon 3, purple/green = upstream/downstream sequences)

ATGGGATATATCCCGGTGCATTTTGTAAGTAGGCCTTATTTTGAAAATGAGGCAATAATGAAAAACTGTGGCCTCAATACAAGAGTCATATTCTACTAAAAATAGGATGGACTTCATAATACGCTAGAGAATCGGGTATGTTAATACACTAGCCTCTTTTGGGTTTTGAGGTTTGCAAACCTATTTGTTATAAAAACTGACCCATTAACAGCCTCTGCTTCATAAATGTTACTTTTTAAAACTCAGTTCGTGGTGGGCTAGAGAGAGAGAGTTCAGAGGTTAGGAACACTGACTGCTCTCCCAGAGGTCCTGAGTTCAATTCCCAGCAACCCATATGGTGGCTCACAACCATCTGTAATGGGATCCGATGCCCTCTTCTGGTGTGTCTGAAGGGAGCGACAGCAGTACTGCATAACACTGGCTGTCTTAGAACTGTGTGCCAAGGCTGGCCTTAAACTCACAGACCGCCTGCCTCTGCCTCCCAAGGGCTGGAAGCAAAGGAGTGCGCCACTCTGATTAACCAACTACTGTACAGGGCTTAAAGCAAGTGTAGCTTATTTAAGGTATCAGTTTGTTTTTTAAAGCCAGGCTTGGCAGCTCATGCCATTTCTTCCAGCATTCAGGAGGCAGAGAGAGGCAGAGCTCTCTGAGTTCAAGGCCAGCCTGGTCTACATGTGTTTAGCATTTAGTGGATGGCAAGGCTTAAAGACAGTTTAATCCCTTCATATTAGACTTTTCCCAGTTAGATCTCTTTGGCTAAGAAAATAAAGGCTTTGGTTTGGTTTTGTCTTTTTTTTTTTTTTTTTTTTTTTTTTTTTTTTTTTACTTTGGAGGCAGTGTGACCTAGTCCCTTCTTAGGGTAGAAGGCTTTCTGAAGGAGCCTAATCCTATAGTGTTTTCTTCTGTCTCTGCAATTTTCTTAGCTAGAACTTTTTTTTAAAACTTTCTAACTTGCATTTTTCCCTACTTTTCCAGGATAGTGTTGATCAAGAACTCCCTTCTTCTCCAAAGCAAACCGTTGGTTTTGATAAGCAGTCAACATTAAAAAAAAGTATGTATATACCTAATATATTTTCATCGTAAACTTGGTTGTTCTTGGAAATGGACCTATGGTAGTGTTTGGCATTTGATAAATATTTCAAGTTTTTCTTTTCAAAGTAAAGTACTTAAAATTTGTTTTTGTTTTTTTTTTTTTTTGTTTTTTGAGGCAGGGTTTCTCTGTGTAGCCCTGGCTATCCTGGAACTCACTCTGTAGACCAGGTTGGCCTCGAACTCAGAAATCCGCCTGCCTCTGCCTCCCGAGTGCTGGGATTAAAGGCATGCGCCACCACGCCCGGCTCTTGTTTTTTTTTTTAAACATAAAACTTTGATGCTGCTTTGCATGAAATATATGAGATTGACTGTCTGCCTCAGTTGGTCCAAAACCAGGAGCACTGGTTTTTCGGGTTTTAATGATATATTTTATAAAAGCTGCAAGGGCAGATTTGGTGGTTCATCCCTTTAATCCTAGCACCCCAGAGGTAGGTGAATCTCTGGGCATTCAAGATGAACCTGGTCTACATAGTAAAGTTCTAGGACAGCCAAGAATTCACAGAGAAACCCTGTCTCAAAAACATAAAATAAATCCGAATCTTTGCTTTTTAAAAATGGTAAAACAATTATCTGACAAATGCAGTTACTAAGTTGGTACTTACTGGCTAAGTTGGCTTGAAGAGATAATTATATGCTTTACGAACAGCTAAGAAGCAAAGGTACACCCGGCCTTGACTTTAATTCCCTCAGAAAAAGTACGTTATTAAGTATTTTTCCTTCGCTTACACAAGTGGTTCTGCTCTGTCGTGAA

**Mutant founder line** (purple/green = upstream/downstream sequences)

ATGGGATATATCCCGGTGCATTTTGTAAGTAGGCCTTATTTTGAAAATGAGGCAATAATGAAAAACTGTGGCCTCAATACAAGAGTCATATTCTACTAAAAATAGGATGGACTTCATAATACGCTAGAGAATCGGGTATGTTAATACACTAGCCTCTTTTGGGTTTTGAGGTTTGCAAACCTATTTGTTATAAAAACTGACCCATTAACAGCCTCTGCTTCATAAATGTTACTTTTTAAAACTCAGTTCGTTCTAGGACAGCCAAGAATTCACAGAGAAACCCTGTCTCAAAAACATAAAATAAATCCGAATCTTTGCTTTTTAAAAATGGTAAAACAATTATCTGACAAATGCAGTTACTAAGTTGGTACTTACTGGCTAAGTTGGCTTGAAGAGATAATTATATGCTTTACGAACAGCTAAGAAGCAAAGGTACACCCGGCCTTGACTTTAATTCCCTCAGAAAAAGTACGTTATTAAGTATTTTTCCTTCGCTTACACAAGTGGTTCTGCTCTGTCGTGAA
